# *Drosophila* Clu ribonucleoprotein particle dynamics rely on the availability of functional Clu and translating ribosomes

**DOI:** 10.1242/jcs.263730

**Published:** 2025-05-09

**Authors:** Hye Jin Hwang, Kelsey M. Sheard, Rachel T. Cox

**Affiliations:** ^1^ Department of Biochemistry and Molecular Biology, Uniformed Services University, Bethesda, MD 20814, USA; ^2^ Henry M. Jackson Foundation for the Advancement of Military Medicine, Inc., Bethesda, MD 20817, USA

**Keywords:** Clu, *Drosophila*, Ribonucleoprotein, Cycloheximide, Translation, Mitochondria

## Abstract

*Drosophila* Clu is a conserved multi-domain ribonucleoprotein essential for mitochondrial function that forms dynamic particles within the cytoplasm. Unlike stress granules and processing bodies (P-bodies), Clu particles disassemble under nutritional or oxidative stress. However, it is unclear how disrupting protein synthesis affects Clu particle dynamics, especially given that Clu binds mRNA and ribosomes. Here, we capitalize on *ex vivo* and *in vivo* imaging of *Drosophila* female germ cells to determine what domains of Clu are necessary for Clu particle assembly and how manipulating translation affects particle dynamics. Using domain deletion analysis, we identified three domains of Clu essential for particle assembly. We also demonstrated that overexpressing functional Clu led to disassembly of particles. In addition, we inhibited translation using cycloheximide and puromycin. In contrast to P-bodies, cycloheximide treatment did not disassemble Clu particles yet puromycin treatment did. Surprisingly, cycloheximide stabilized particles under oxidative and nutritional stress. These findings demonstrate that Clu particles display novel dynamics in response to altered ribosome activity and support a model where they function as translation hubs whose assembly heavily depends on the dynamic availability of translating ribosomes.

## Teaser

**Summary:** The dynamics of Clu ribonucleoprotein particles are governed by its functional protein level and translating ribosomes, giving insight into how these unique particles regulate mitochondrial biology in *Drosophila*.

## INTRODUCTION

Ribonucleoproteins (RNPs) often associate with cytoplasmic particles or bodies to form RNP particles, which play crucial roles in the post-transcriptional regulation of mRNAs (Gehring et al., 2017). These RNP particles, including stress granules and processing bodies (P-bodies), are highly conserved across species and function to sequester translation machinery and mRNAs, thereby regulating mRNA stability or active translation (Anderson and Kedersha, 2006; Bauer et al., 2023; Kato and Nakamura, 2012; Keene, 2007). Under normal conditions, stress granules and P-bodies are present in limited quantities, but their numbers increase significantly in response to cellular stress (Lin et al., 2008; Schisa, 2019).

**Fig. 1.**
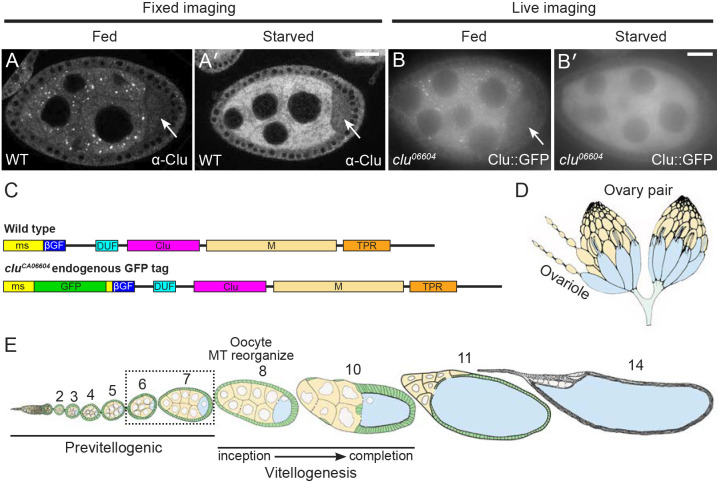
**Clu forms abundant cytoplasmic particles in *Drosophila* nurse cells.** (A–B′) Stage 7 follicles. Clu protein visualized in fixed follicles (A,A′) and still frames from live-imaged follicles (B,B′). The differences in tissue clarity are due to immunostaining (A,A′) versus live imaging (B,B′). Large cytoplasmic Clu particles are present in well-fed flies (A,B). Particles are disassembled upon starvation (A′,B′). Clu protein is reduced in the oocyte (A,B, arrows) compared to the nurse cells, and particles are absent (Sheard et al., 2020). Fixed images were obtained using a Zeiss 700 confocal laser scanning microscope (Carl Zeiss Microscopy LLC, White Plains, NY, USA), and live images were obtained using a Nikon Eclipse Ti2 spinning disk microscope with a 100× objective lens (Nikon Corporation, Tokyo, Japan). See Sheard et al., 2020 for details. (C) Schematic showing Clu protein domains. *clu^CA06604^* has an in-frame GFP inserted in the endogenous *clu* locus, resulting in a GFP fusion protein (Clu::GFP). ms, *melanogaster* specific; βGF, β-grasp fold; DUF, domain of unknown function; M, middle domain; TPR, tetratricopeptide repeat. (D,E) Schematics depicting *Drosophila* oogenesis (Hwang and Cox, 2024). Female *Drosophila* have a pair of ovaries (D) composed of strings of developing follicles called ovarioles (E). (E) Modified from Cummings and King (1969). Ovaries from well-fed females contain all the developing follicle stages (stages 2–14). Follicles are composed of 15 nurse cells (yellow) and one oocyte (blue) surrounded by somatic follicle cells (green). Vitellogenesis starts at stage 8 when the polarity of the oocyte's microtubule (MT) cytoskeleton changes. Analysis and images presented in this study are predominantly stages 6 and 7 (dashed box). White, anti-Clu antibody (A,A′) and GFP (B,B′). Scale bars: 20 µm.

*Drosophila* Clueless (Clu) and its vertebrate analog CLUH are highly conserved multidomain ribonucleoproteins abundantly found in the cytoplasm (Fig. 1A–C; Cox and Spradling, 2009; Gao et al., 2014; Sen and Cox, 2016). Clu forms robust particles *in vivo*, especially in female germ cells, which exhibit high metabolic activity (Fig. 1A,B; Cox and Spradling, 2009; Sheard et al., 2020). The loss of Clu/Cluh in *Drosophila* and mice leads to profound mitochondrial dysfunction, with flies living only a few days and mice dying on postnatal day 1 (Cox and Spradling, 2009; Schatton et al., 2017). *clu* mutants also have reduced mitochondrial proteins (Sen and Cox, 2022). Studies of CLUH have shown that a significant portion of CLUH-bound transcripts encode nucleus-encoded mitochondrial proteins, suggesting that CLUH is involved in the regulation of mRNAs that are crucial for mitochondrial function (Gao et al., 2014). *Drosophila* Clu also binds transcripts encoding mitochondrial proteins (Sen and Cox, 2022). The mechanism through which Clu/CLUH regulates these associated mRNAs is not yet fully understood (Hémono et al., 2022a; Schatton et al., 2017; Vardi-Oknin and Arava, 2019). Moreover, the association of Clu with ribosomal proteins and translation factors suggests that it plays a role in active translation, potentially involving mitochondria-associated ribosomes for co-translational or site-specific import, as well as non-mitochondria-associated cytoplasmic ribosomes (Hémono et al., 2022a; Vardi-Oknin and Arava, 2019; Bennett et al., 2022; Pla-Martin et al., 2020; Sen and Cox, 2022).

In *Drosophila*, Clu forms mitochondria-associated particles of various sizes that are highly dynamic and require intact microtubules for movement (Sheard et al., 2020). Although Clu self-associates, it remains unclear whether Clu forms multimers or simply aggregates within these particles (Sen and Cox, 2016). These particles, which we have named ‘bliss particles’, do not colocalize with common subcellular organelle markers such as the autophagosome marker Atg8 or the P-body protein Trailer Hitch (Sheard et al., 2020). Unlike stress granules and P-bodies, Clu particles are exquisitely sensitive to stress *in vivo* and only form under optimal conditions in well-fed flies (Sheard et al., 2020). Particle disassembly can be induced by starvation, oxidative stress and mitochondrial stress caused by mutations in *Superoxide Dismutase 2* (*Sod2*), *PTEN-induced putative kinase 1* (*Pink1*) and *parkin* (Fig. 1A′,B′; Cox and Spradling, 2009; Sheard et al., 2020; Sen et al., 2015). Remarkably, removing stress conditions, such as by refeeding the flies or by adding insulin *ex vivo*, restores Clu particles (Sheard et al., 2020). Despite the dynamic nature of these particles, Clu levels remain constant, indicating that Clu particle disassembly is not due to protein degradation (Sheard et al., 2020). Furthermore, insulin signaling is necessary and sufficient for particle assembly, suggesting that the metabolic state of the cell significantly influences the presence of Clu particles (Sheard et al., 2020).

Mitochondria, as central hubs of metabolism, house critical pathways including heme biosynthesis, fatty acid β-oxidation and steroidogenesis, in addition to generating ATP (Bennett et al., 2022; Rahman, 2020; Scarpulla, 2008). Although these organelles have their own mitochondrial DNA, the majority of proteins required for these pathways are supplied by nuclear DNA-encoded mRNAs that are translated on cytoplasmic and mitochondrial-associated ribosomes (Bennett et al., 2022; Devaux et al., 2010; Dimmer et al., 2002). Given binding of Clu to mRNAs and its association with ribosomes, as well as the juxtaposition of Clu particles to mitochondria, this study aims to determine how disrupting translation affects Clu particle dynamics in *Drosophila* female germ cells. We employed ectopic transgenic expression *in vivo* to demonstrate that three conserved domains of Clu are necessary for its assembly into particles and that an excess of full-length, but not deletion constructs of Clu disassemble particles, suggesting particle stability or assembly is altered with non-physiological Clu levels. Manipulating translation activity using the translation inhibitors puromycin (PUR) and cycloheximide (CHX) revealed that PUR treatment quickly led to disassembly of Clu particles whereas CHX treatment did not. In addition, CHX treatment stabilized existing Clu particles in the presence of nutritional and oxidative stress. Together, these observations support a model whereby *Drosophila* Clu particle assembly requires translating ribosomes and that particles could function as sites of active translation of mRNAs encoding mitochondrial proteins to sense changes to cellular metabolism and subsequently regulate mitochondrial function. In addition, our observed particle dynamics are unique from those observed for other RNP particles, underscoring the distinctive response of Clu particles to translation inhibition in *Drosophila* germ cells.

## RESULTS

### *Drosophila* ovaries as a model to study Clu particle dynamics

To identify the protein domains and ribosome activity requirements of Clu particle dynamics, we examined particle assembly and disassembly in female germ cells using fixed and live imaging (Fig. 1A–B′). Clu particles are always present in the nurse cells of follicles from well-fed flies (Fig. 1A,B; Cox and Spradling, 2009; Sheard et al., 2020). However, they look larger or smaller, or more or less numerous, depending on imaging conditions, including the length of dissection, whether the tissue is imaged while fixed or live, and the type of microscope (laser confocal versus spinning disc). Also, importantly, Clu particles completely disassemble in response to stress (Fig. 1A′,B′); thus we used a binary decision for assembly or disassembly – either Clu particles were present or absent – to determine how various conditions affect dynamics. *Drosophila* females have a pair of ovaries composed of 16–20 ovarioles (Fig. 1D,E; Spradling, 1993; Sarikaya et al., 2012). Ovarioles are strings of developing follicles that contain all follicle stages (2–14) when dissected from well-fed females. For consistency, we predominantly analyzed data from stage 6 and 7 follicles, which are large enough to be readily imaged and contain many Clu particles but are previtellogenic and have not yet undergone many complex developmental events (Fig. 1E).

### The DUF, Clu and TPR domains are necessary for Clu particle association

Clu is a large, multi-domain protein (Fig. 1C); however, the function of each domain and how they controls particle dynamics is poorly understood. The melanogaster-specific (ms) domain is unique to *Drosophila* and is not required to rescue the *clu* null mutant (Sen and Cox, 2016). The β-grasp fold (βGF) domain is so-called based on the predicted structure, and the domain of unknown function (DUF) is predicted based on sequence homology (Sen et al., 2015). The Clu domain is highly conserved, but we do not yet understand its role. We have previously demonstrated that the tetratricopeptide repeat (TPR) domain is crucial for Clu to bind mRNA (Sen and Cox, 2016). Finally, the middle (M) domain sequence is unstructured, which is a feature commonly found in proteins associated with membraneless organelles (Shin and Brangwynne, 2017; Alberti et al., 2019; Li et al., 2012).

**Fig. 2.**
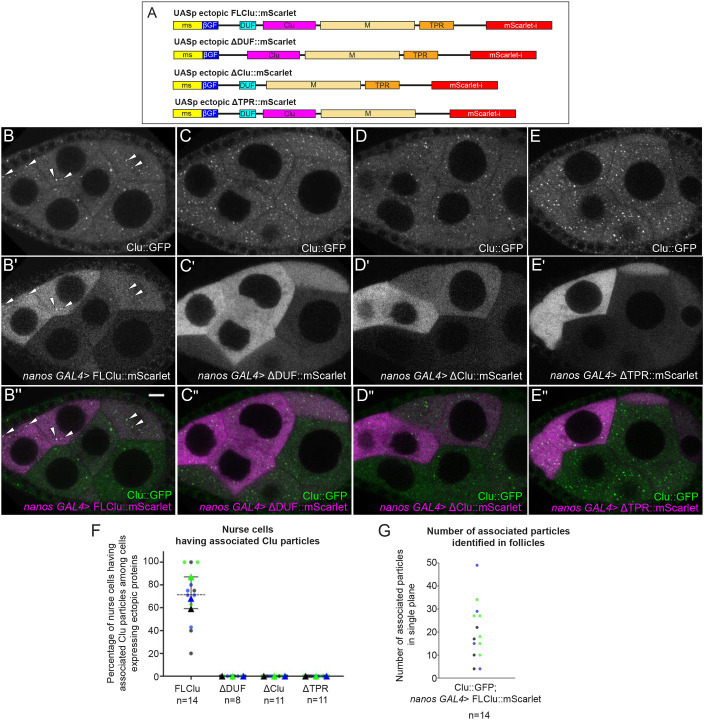
**DUF, Clu and TPR domains are required for Clu particle association.** (A) Cartoon of full-length (FL) and domain deletion (ΔDUF, ΔClu and ΔTPR) constructs of ectopic Clu tagged with mScarlet. (B–B″) Still images from Movie 1 of a follicle from a *clu^CA06604^*/*+*; *nanos (nos) GAL4*/*UASp-FLclu*::*mScarlet* female. Particles from endogenous Clu::GFP (B) and ectopic FLClu::mScarlet (B′) colocalize in germ cells (B″), as arrowheads indicate (see Fig. S2A,A′ for details). 71% of nurse cells expressing mScarlet showed colocalization of Clu::GFP and mScarlet (F, FLClu). (C–E″) Still images from ectopic Clu deletion constructs (Movies 2–4). Endogenous Clu::GFP (C,D,E) assembles particles. However, ΔDUF (C′), ΔClu (D′), and ΔTPR (E′) constructs do not assemble particles (C′,D′,E′) nor do they associate with endogenous Clu particles (C″,D″,E″, F). Still images from Movie 2 (C–C″) of a follicle from *clu^CA06604^*/+*; nosGAL4*/*UASp-clu*Δ*DUF*::*mScarlet*, Movie 3 (D–D″) of a follicle from *clu^CA06604^*/+*; nosGAL4*/*UASp-clu*Δ*Clu*::*mScarlet*, and Movie 4 (E–E″) of a follicle from *clu^CA06604^*/+; *nosGAL4*/*UASp-clu*Δ*TPR*::*mScarlet* females. (B–E″) Stage 7 egg chamber follicles expressing Clu::GFP and various ectopic Clu tagged with mScarlet were imaged with a 200 µg/ml insulin-containing CS medium in a time-lapse course at a single plane (see Movies 1–4 for details). The focal plane was selected to ensure more than three nurse cells having nuclear and cytosolic area clearly were visible, with ∼25% depth from the top surface of each follicle (See Materials and Methods for details). Live images were obtained using a Nikon A1 plus Piezo Z Drive Confocal microscope with a 60× objective lens (Nikon Corporation, Tokyo, Japan). (F) The graph indicates the percentage of the nurse cells having colocalization of endogenous Clu::GFP and ectopic mScarlet among the nurse cells expressing mScarlet within an egg chamber. (G) The graph indicates the number of the identified colocalized Clu::GFP and mScarlet particles in a single frame of each egg chamber analyzed in (F, FLClu as shown in Fig. S2A,A′). In F and G, circles represent individual egg chamber analyzed, with the color showing the independent experimental group. In F, triangles shows average mean of each color set. *n*, total follicle number analyzed. Error bars are s.e.m. (B,C,D,E) White, endogenous Clu::GFP. (B′,C′,D′,E′) White, mScarlet. (B″,C″,D″,E″, merge) Green, Clu::GFP; magenta, mScarlet. Scale bar: 10 µm.

Previously, we used co-immunoprecipitation to show that full-length *Drosophila* Clu can self-associate (Sen and Cox, 2016). To test which domains are required for Clu particle assembly *in vivo*, we used the GAL4/UAS system (Brand and Perrimon, 1993). We created transgenic lines that ectopically express Clu-tagged with mScarlet at the C-terminus under the control of the conditional UASp promoter (Fig. 2A). Each construct was expressed at the appropriate molecular mass (Fig. S1A,B). *clu* null mutants are weak and infertile with very small ovaries, and die after ∼7 days (Cox and Spradling, 2009). Expressing full-length (FL)-Clu::mScarlet ubiquitously, rescued the *clu* null mutant, producing healthy, fertile females (Fig. S1C). We visualized endogenous Clu using GFP inserted at the *clu* locus (*clu^CA06604^*, Clu::GFP; Fig. 1C) (Buszczak et al., 2007). To simultaneously visualize ectopically expressed mScarlet-labeled constructs and endogenous Clu, each construct was combined with germline-specific *nanos* GAL4 (*nos*GAL4) in a *clu^CA06604^* background (Van Doren et al., 1998). The *nos*GAL4 line we used is clonally expressed in the nurse cells (Rorth, 1998). Using live imaging, we found that full-length (FL)-Clu::mScarlet reliably formed Clu particles (Fig. 2B′,F,G), which colocalized with endogenous Clu::GFP particles, indicating that both Clu species exist within the same particle (Fig. 2B″; Fig. S2A,A′, Movie 1). To determine whether the DUF, Clu, and TPR domains are required for particle assembly, we ectopically overexpressed mScarlet-labeled Clu transgenes with each domain deleted (ΔDUF::mScarlet, ΔClu::mScarlet and ΔTPR::mScarlet) (Fig. 2A,C–E″, Movies 2–4). Using live imaging, we were unable to see particle assembly for any of these deletion constructs (Fig. 2C′,D′,E′,F). Furthermore, none of these deletions co-labeled with endogenous Clu::GFP particles (Fig. 2C″,D″,E″; Fig. S2B–D′). This suggests that these three domains are necessary to assemble Clu particles and to associate with endogenous particles that had already assembled.

### Clu particles disassemble in response to high expression of functional Clu

**Fig. 3.**
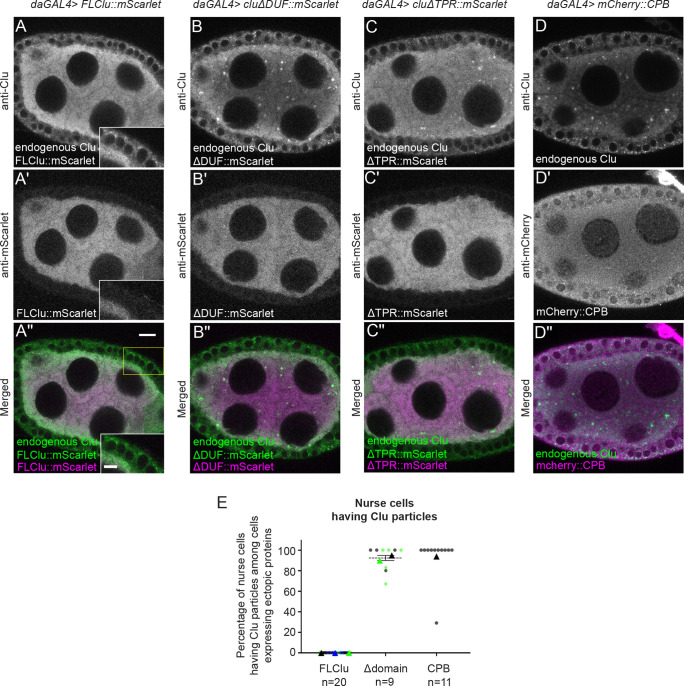
**High levels of functional Clu disassemble Clu particles.** (A–A″) Immunostaining of a follicle from a *daughterless* (*da*) *GAL4*/*UASp-FLclu*::*mScarlet* female. High levels of ectopic FLClu (A′) lead to disassembly of endogenous particles (A,A″). Particles assemble in the surrounding somatic cells (A,A″, insets). (B–B″) Immunostaining of a follicle from a *daGAL4*/*UASp-clu*Δ*DUF*::*mScarlet* female. (C–C″) Immunostaining of a follicle from a *daGAL4*/*UASp-clu*Δ*TPR*::*mScarlet* female. (D–D″) Immunostaining of a follicle from *daGAL4*/*UASp-mCherry*::*cpb* female. High levels of ΔDUF (B′), ΔTPR (C′) or CPB (D′) do not interfere with endogenous Clu particle assembly (B,B″ for ΔDUF, C,C″ for ΔTPR or D,D″ for CPB). (A–D″) Stage 7 egg chamber follicles were imaged with a 1.2 µm thickness of *z*-stacks with an interval of 0.42 µm. The focal plane was selected by ensuring at least three to four nuclei were clearly visible in nurse cells, but also to avoid dim fluorescence signals due to deeper depth (see Materials and Methods for details). Images were obtained using a Zeiss LSM 980 confocal laser scanning microscope (Carl Zeiss Microscopy). (E) 95% (ΔDUF, Δdomain black), 90% (ΔTPR, Δdomain green) and 94% (CPB) of nurse cells expressing each ectopic construct showed Clu particles. (E) Circles indicate results for individual egg chambers analyzed; triangles show the average mean of each color set. Colors represent different independent experimental groups for FLClu or different Δdomain group, *n*: total follicle number analyzed. (A,B,C,D) White, anti-Clu. (A′,B,′C′) White, anti-mScarlet. (D′) White, anti-mCherry. (A″,B″,C″, merge) Green, anti-Clu; magenta, anti-mScarlet. (D″, merge) Green, anti-Clu; magenta, anti-mCherry. For A,A″,B,B″,C,C″, note that the anti-Clu antibody also recognizes the mScarlet transgene*.* Scale bar: 10 µm (main images); 5 µm (insets).

When we tested expression levels of ectopic FL-Clu using different GAL4 drivers, we noticed a dose-dependent effect on particle assembly. *daughterless* (*da*) GAL4 is a ubiquitous GAL4 driver that when combined with FL-Clu::mScarlet rescued the *clu* null mutant (Fig. S1C) (Wodarz et al., 1995). *da*GAL4 induced high uniform expression of Clu in germ cells in contrast to lower expression levels of *nos*GAL4, consistent with *da* expression pattern (Fig. 3A–A″; Figs S1A, S3A–D″) (Cummings and Cronmiller, 1994). When ectopic FL-Clu::mScarlet was expressed using *da*GAL4 (Fig. 3A′,E), neither ectopic Clu nor endogenous Clu assembled into particles in germ cells (Fig. 3A versus Fig. S3D). Mitochondrial distribution remained normal, indicating that the germ cells were not experiencing a stress to cause mislocalization (Fig. S3F′ versus E′ control). We also observed that endogenous Clu assembled into particles in the somatic follicle cells, where *da*Gal4-induced ectopic Clu expression was lower compared to the germ cells (Fig. 3A,A″, insets; Fig. S3B,B″). To test whether this was dependent on functional FL-Clu, we ectopically overexpressed a Scarlet-labeled ΔDUF or ΔTPR domain (ΔDUF::mScarlet, ΔTPR::mScarlet, Fig. 2A) using *da*GAL4. Consistent with our observations using *nos*GAL4, neither was able to mediate assembly of mScarlet particles (Fig. 3B′,C′), but unlike ectopic FL-Clu, ΔDUF and ΔTPR overexpression did not lead to disassembly of endogenous Clu particles (Fig. 3B,C). Finally, to ensure that expressing high concentrations of any protein does not cause cell stress that inhibited particle assembly, we sought an unrelated cytoplasmic protein. We used mCherry-labeled Capping Protein β(mCherry::CPB). CPB is a well-characterized and abundant actin-binding protein (Ogienko et al., 2013), and mCherry and mScarlet are highly similar monomeric red fluorescent proteins derived from *Discosoma* sp. (Bindels et al., 2017; McCullock et al., 2020; Shaner et al., 2004). mCherry::CPB also did not disrupt endogenous Clu particles (Fig. 3D–D″). This suggests that the mechanism assembling Clu particles depends on regulating the concentration of functional Clu but does not respond to non-functional Clu (ΔDUF and ΔTPR).

### The translation inhibitor puromycin disassembles Clu particles

**Fig. 4.**
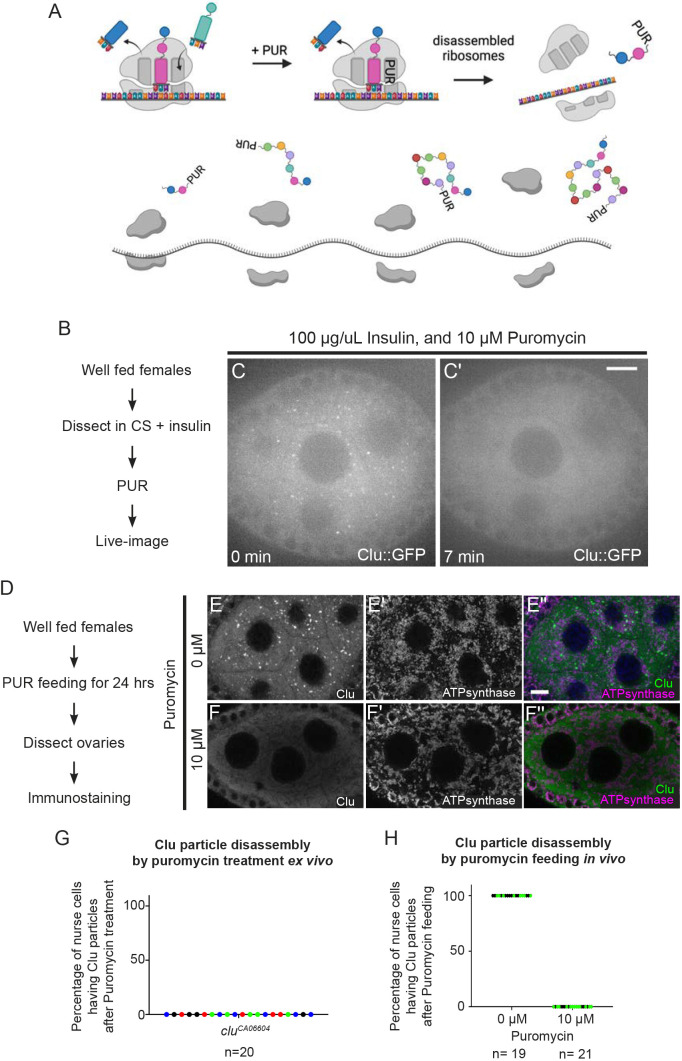
**The translation inhibitor puromycin disassembles Clu particles.** (A) Schematic illustrating the mechanism of action for the translation inhibitor puromycin (PUR). PUR blocks nascent polypeptide chain elongation, thereby causing premature translation termination, disassembly of the ribosomal complex, and decreased polysomes. Created in BioRender by Cox, R., 2025. https://BioRender.com/82399h9. This figure was sublicensed under CC-BY 4.0 terms. (B) Workflow for the experiment shown in C,C′. Ovarioles dissected from well-fed *clu^CA06604^* females were treated with PUR by injection to the medium, then live imaged (C,C′; Movie 5). (C) The first still frame (at time zero after adding 10 µM PUR) of stage 6 follicle from Movie 5 showing Clu particles. (C′) The 22nd still frame (at 7 min) of the same follicle, demonstrating Clu particles disassembly upon with PUR treatment (G, *n*=20/20 follicles). The focal plane was selected by ensuring at least three to four nuclei were clearly visible in nurse cells, with ∼25% depth from the top surface of each follicle (see Materials and Methods for details). (D) Workflow for the experiment shown in (E–F″). Well-fed *w^1118^* females were fed with the wet yeast paste containing (E–E″) 0 µM or (F–F″) 10 µM PUR for 24 h before dissection, followed by immunostaining for Clu and mitochondria (ATP synthase). (F–F″) Stage 7 follicles from females fed for 24 h with wet yeast paste containing 10 µM PUR had disassembled Clu particles (F,F″) but maintained normal mitochondrial morphology and localization (F′,H, second column, *n*=21/21 follicles), whereas the follicles from females fed no PUR maintained Clu particles (E,E″,H, first column, *n*=19/19 follicles). Images are 1.2 µm projections assembled from 0.42 µm sections. The focal plane was selected to show at least three to four nuclei but also to avoid dim fluorescence signals due to deeper depth (see Materials and Methods for details). (G) The graph indicates the percentage of follicles with disassembled Clu particles after puromycin treatment in B–C′. (H) The graph indicates the percentage of follicles that had Clu particles after 24 h of PUR feeding in D–F″. In G and H, circles indicate results for individual egg chambers analyzed. Colors represent different independent experimental groups. *n*: total follicle number analyzed. Live images were obtained using a Nikon Eclipse Ti2 spinning disk microscope with a 100× objective lens (Nikon Corporation). Immunostaining images were obtained using a Zeiss LSM 980 confocal laser scanning microscope (Carl Zeiss Microscopy). (E,F) White=anti-Clu. (E′,F′) White, anti-ATP synthase. (E″,F″, merge) Green, anti-Clu; magenta, anti-ATP synthase. Scale bars: 10 µm.

Stress granules and P-bodies have distinct assembly–disassembly responses downstream of various stressors and different translation inhibitors, depending on the mechanisms of action for the drugs (Buddika et al., 2020; Eulalio et al., 2007; Patel et al., 2016). This is thought to be due to manipulation of available levels of associated messenger RNPs (mRNPs) and polysome presence and activity (Hofmann et al., 2021; Hubstenberger et al., 2017; Kato and Nakamura, 2012; Ivanov et al., 2019). Puromycin (PUR) is a commonly used and unique translation inhibitor. PUR forms a stable peptide bond with nascent polypeptides, resulting in premature translation termination which leads to ribosome complex disassembly, polysome loss and increased free mRNAs (Fig. 4A) (Aviner, 2020). Given that Clu is a ribonucleoprotein crucial for mitochondrial function, binds mRNAs encoding mitochondrial proteins and associates with the ribosome, we wanted to determine how puromycin treatment affected Clu particle dynamics. To do this, we treated ovarioles dissected from well-fed *clu^CA06604^* females with PUR and found that the plentiful Clu particles were quickly and completely disassembled within 10 min (Fig. 4B–C′,G; Movie 5). *Ex vivo* imaging has many advantages for investigating cellular dynamics. To determine the effect of PUR treatment *in vivo*, we switched well-fed females to PUR mixed in wet yeast paste for 24 h, then used fixed imaging to analyze Clu particles. Feeding females PUR recapitulated our *ex vivo* result, resulting in disassembly of Clu particles (Fig. 4D–F″,H). Given the molecular action of PUR, this suggests that decreased translating ribosomes, disassembled ribosomes and/or increased concentrations of mRNPs cause Clu particles to disassemble.

### Cycloheximide treatment maintains Clu particles, but blocks insulin-induced assembly

**Fig. 5.**
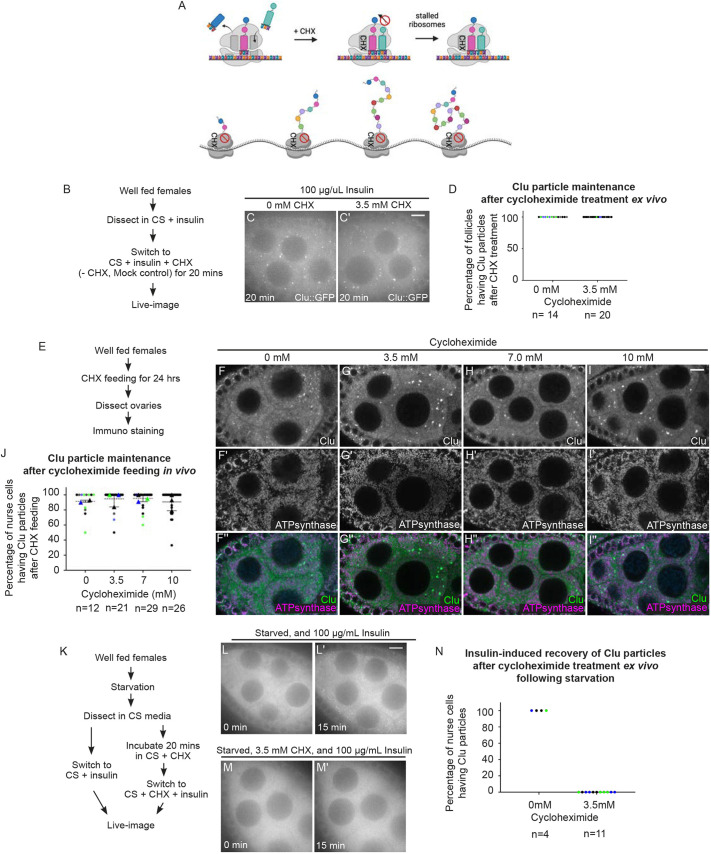
**The translation inhibitor cycloheximide maintains Clu particles, but blocks insulin-induced assembly.** (A) Schematic demonstrating the mechanism of action for the translation inhibitor cycloheximide (CHX). CHX blocks the 60S ribosome subunit exit site, thereby stalling translation and increasing polysome numbers. Created in BioRender by Cox, R., 2025. https://BioRender.com/emtju4m. This figure was sublicensed under CC-BY 4.0 terms. (B) Workflow for the CHX treatment showing in C,C′. Ovarioles from well-fed *clu^CA06604^* females were incubated with CHX for 20 min then live imaged (C,C′; Movies 6,7). (C) Still image of a stage 7 follicle after a 20-min incubation without CHX demonstrating the presence of Clu particles (D, first column, *n*=14/14 follicles). (C′) Still image of a stage 7 follicle after a 20-min incubation with 3.5 mM CHX demonstrating that the assembled Clu particles are still present (D, second column, *n*=20/20 follicles). Follicles were imaged at 3-min intervals at a single plane for 20 min. The focal plane was selected by ensuring at least three to four nuclei were clearly visible in the nurse cells, with ∼25% depth from the top surface of each follicle (see Materials and Methods for details). (D) The percentage of follicles having Clu particles after CHX treatment in C,C′. (E) Workflow for the experiments shown in F–I″. Well-fed *w^1118^* females were fed wet yeast paste containing (F–F″) 0 µM, (G–G″) 3.5 mM, (H–H″) 7 mM CHX, and (I–I″) 10 mM CHX for 24 h before dissection, followed by immunostaining for Clu and mitochondria (ATP synthase). (F–I) CHX feeding did not lead to disassembly of Clu particles and (F′–I′) normal mitochondrial morphology and localization was maintained. (J) Graph indicating the percentage of nurse cells having Clu particles: 91% (0 mM CHX), 94% (3.5 mM CHX), 96% (7 mM CHX), and 92% (10 mM CHX) of nurse cells. Images are 2 µm projections assembled from 0.42 µm sections. The focal plane was selected to show at least three to four nuclei but also to avoid dim fluorescence signals due to deeper depth (see Materials and Methods for details). Error bars are mean±s.e.m. (K) Workflow for the CHX experiments shown in L–M′. Well-fed *clu^CA06604^* females were starved for 3 h with water only, then ovarioles dissected from starved animals were incubated in insulin (L,L′, control) or CHX followed by insulin (M,M′). (L) The first still frame (at time zero after adding 100 µg/ml insulin) of stage 7 follicle from Movie 8 showing no Clu particles. (L′) The 46th still image (at 15 min) of the same follicle from Movie 8 demonstrating the recovery of Clu particles by insulin as previously shown (Sheard et al., 2020) (N, first column, *n*=4/4 follicles). (M) The first still frame (at time zero after adding 100 µg/ml insulin) of stage 7 follicle starved and treated with CHX from Movie 9. (M′) The 46th still frame (at 15 min) of the same follicle showing no insulin-induced Clu particle assembly following CHX treatment (N, second column, *n*=11/11 follicles). The focal plane was selected by ensuring at least three to four nuclei were clearly visible in the nurse cells, with ∼25% depth from the top surface of each follicle (see Materials and Methods for details). In D, J and N, circles indicate individual egg chamber analyzed, color represent different independent experimental group; in J, triangles indicate average mean of each color set. *n*: total follicle number analyzed. Live images were obtained using a Nikon Eclipse Ti2 spinning disk microscope with a 100× objective lens (Nikon Corporation). Immunostaining images were obtained using a Zeiss LSM 980 confocal laser scanning microscope (Carl Zeiss Microscopy). (F,G,H,I) White, anti-Clu. (F′,G′,H′,I′) White, anti-ATP synthase. (F″,G″,H″,I″, merge) Green, anti-Clu; magenta, anti-ATP synthase. Scale bars: 10 µm.

CHX is another well-known and frequently used translation inhibitor that binds to the exit site of the ribosome, which stalls the ribosome and blocks translation elongation, leading to increased concentrations of polysomes (Fig. 5A) (Duncan and Mata, 2017). Under normal conditions, CHX treatment causes P-bodies to disassemble (Eulalio et al., 2007; Lin et al., 2008; Moutaoufik et al., 2014; Patel et al., 2016; Kedersha et al., 2000). To ensure our method of CHX treatment was effective, we dissected ovarioles from well-fed *trailer hitch^CA06517^* (*tral*) females that expressed GFP inserted at the endogenous *tral* locus, thus labeling P-bodies (Kato and Nakamura, 2012; Eulalio et al., 2007; Buszczak et al., 2007). We confirmed that Tral-labeled P-bodies decreased in size and number with CHX addition compared to mock control, as previously shown (Fig. S4; Eulalio et al., 2007; Patel et al., 2016). To determine the effect of CHX on Clu particles *ex vivo*, we tested whether CHX treatment caused disassembly of particles and found that this was not the case (Fig. 5B–D).

To determine the effect of CHX on Clu particles *in vivo*, we switched well-fed females to yeast paste supplemented with CHX for 24 h. Feeding CHX reduces protein synthesis (Hwang and Cox, 2024). To determine the effect of dietary CHX on Clu particle dynamics *in vivo*, we fixed and immunolabeled ovarioles from CHX-fed females (Fig. 5F–I″). As expected, untreated well-fed females displayed robust Clu particles (Fig. 5F,F″). Like our *ex vivo* experiment, CHX-fed females also exhibited robust Clu particles, indicating that CHX feeding does not disassemble particles (Fig. 5G,G″,H,H″,I,I″,J). We previously demonstrated that mitochondria mislocalize in nurse cells when the females are exposed to various stressors (Cox and Spradling, 2009; Sen and Cox, 2016; Sen et al., 2015; Sheard et al., 2020). CHX-feeding not only maintained Clu particles, but also maintained mitochondrial morphology and distribution, supporting that feeding CHX did not cause stress signals sufficiently acute to cause mitochondrial mislocalization in nurse cells (Fig. 5F–I″).

Given that CHX did not disassemble Clu particles *ex vivo* and *in vivo*, we wanted to test the effect of CHX on particle assembly. To do this, we dissected ovaries from starved *clu^CA06604^* females, which have completely disassembled Clu particles (Fig. 5K–M′; Movies 8,9) (Sheard et al., 2020). Normally, adding insulin to the medium causes Clu particles to quickly assemble (Fig. 5L,L′,N; Movie 8; Sheard et al., 2020). However, with preincubation of CHX, insulin-induced Clu particle assembly was blocked (Fig. 5M,M′,N; Movie 9). Taken together, these data suggest that the stalled ribosomes occurring with CHX treatment do not lead to the disassembly of Clu particles that have already formed. In addition, insulin signaling is insufficient to drive particle assembly in the absence of pre-existing particles after CHX treatment, when ribosomes are stalled.

### Cycloheximide maintains Clu particles in the presence of nutritional stress

**Fig. 6.**
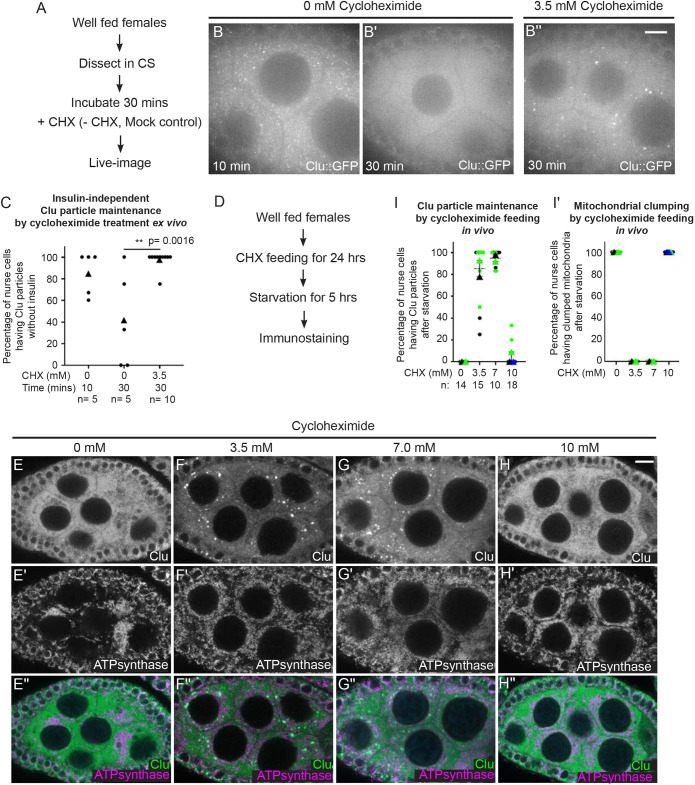
**Cycloheximide maintains Clu particles in the presence of nutritional stress.** (A) Workflow for the experiment. Ovarioles from well-fed *clu^CA06604^* females were dissected without insulin, then treated with 3.5 mM CHX (B″) or not (mock treatment, B,B′). (B) Still image of stage 7 follicle from well-fed females showing that Clu particles are maintained without insulin for 10 min (C, first column, *n*=5 follicles, 85% of the nurse cells have Clu::GFP particles), (B′) but the Clu particles are lost after 30 min of incubation (C, middle column, *n*=5 follicles, 42% of nurse cells have Clu::GFP particles). (B″) Still image of stage 7 follicle treated with 3.5 mM CHX for 30 min demonstrating CHX treatment did not cause particle dispersion even without insulin (C, third column, *n*=10 follicles, 98% of nurse cells have Clu::GFP particles). The focal plane was selected to show at least three to four nuclei were clearly visible in the nurse cells, with ∼25% depth from the top surface of each follicle (see Materials and Methods for details). (C) The graph indicates that the effect of CHX on Clu particle maintenance is significant (**P=0.0016, unpaired two-tailed *t*-test). (D) Workflow for the experiment shown in E–H″. Well-fed *w^1118^* females were fed wet yeast paste containing (E–E″) 0 mM, (F–F″) 3.5 mM, (G–G″) 7 mM CHX or (H–H″) 10 mM CHX and then starved for 5 h. Ovaries were dissected and immunostained for Clu and mitochondria (ATP synthase). (E–H″) Immunolabeled stage 7 follicles showed starvation induced particle disassembly (E) and caused mitochondrial clustering (E′) as we have previously shown (Sheard et al., 2020). (F–F″) 3.5 mM and (G–G″) 7 mM CHX feeding maintained assembled Clu particles (F,G) and normal mitochondrial localization (F′,G′) despite starvation. (H–H″) 10 mM CHX feeding could not maintain the assembled Clu particles (H) and normal mitochondrial morphology (H′) under nutritional stress. 87% (3.5 mM CHX following starvation, I, second column, *n*=15 follicles) and 94% (7 mM CHX following starvation, I, third column, *n*=14 follicles) of nurse cells from the observed follicles had Clu particles (I). (I,I′) The graph indicates that percentage of nurse cells having Clu particles (I) or mitochondrial clumping (I′) under each experimental condition. Error bars are mean±s.e.m. Images are 2 µm projections assembled from 0.42 µm sections. The focal plane was chosen to show greater than three nurse cells, maintaining clear visibility for nuclear and cytoplasmic area, and avoiding dim fluorescence signals due to deeper depth (see Materials and Methods for details). (E,F,G,H) White, anti-Clu. (E′,F′,G′,H′) White, anti-ATP synthase. (E″,F″,G″,H″, merge) Green, anti-Clu; magenta, anti-ATP synthase. In C, I and I′, circles indicate individual egg chamber analyzed, triangles indicate average mean of each color set. Colors show different independent experimental group. *n*: total follicle number analyzed. Scale bars: 10 µm.

For effective *Drosophila ex vivo* imaging, insulin must be added to the medium to fully support the tissue (Morris and Spradling, 2011). If it is omitted, egg chamber development is not normal, and the samples start to degenerate (Prasad and Montell, 2007; Prasad et al., 2007). Given that *Drosophila* Insulin-like peptides secreted by the brain are required for normal egg chamber development, incubating follicles without insulin does not supply effective nutritional signaling and is stressful to the tissue (LaFever and Drummond-Barbosa, 2005; Richard et al., 2005). Given that CHX treatment did not abolish Clu particles, in contrast to PUR, we tested whether CHX treatment could confer a protective effect to maintain Clu particles in the presence of nutritional stress (Fig. 6A–C). Ovarioles dissected from well-fed *clu^CA06604^* females and incubated in insulin-free medium (Complete Schneider's; CS) showed disassembly of Clu particles within 30 min, supporting the crucial role of nutrition in maintaining particles (Fig. 6B,B′). Surprisingly, adding CHX after dissection was sufficient to maintain Clu particles for 30 min, even incubating in the absence of insulin (Fig. 6B″,C). To test whether CHX could also protect Clu particles from nutritional stress *in vivo*, we switched well-fed females to yeast paste supplemented with CHX for 24 h, then subjected the flies to a mild 5-h starvation on water only (Fig. 6D). The 5-h starvation led to completely disassembly of Clu particles (Fig. 6E,E″) (Sheard et al., 2020). However, providing CHX for 24 h before starvation was sufficient to maintain Clu particles and protect them from disassembly (Fig. 6F,F″,G,G″,I). This was not the case with the highest concentration of CHX (Fig. 6H,H″). Although 10 mM CHX feeding did not affect Clu particles under well-fed conditions (Fig. 5I,I″), adding nutritional stress led to disassembly of the particles (Fig. 6H,H″,I). With 3.5 mM and 7.0 mM feeding, not only were particles maintained, CHX feeding before starvation appeared to decrease cellular nutritional stress as indicated by normal mitochondrial localization (Fig. 6E′ versus F′,G′,I′) (Sheard et al., 2020). In contrast, 10 mM feeding caused mitochondrial clumping (Fig. 6H′,H″,I′). This observation supports that stalled ribosomes maintain and protect Clu particles and mitochondrial distribution even with decreased nutrition *ex vivo* and *in vivo*, but that this protection becomes ineffective with a high dose of CHX.

### Cycloheximide maintains Clu particles in the presence of oxidative stress

**Fig. 7.**
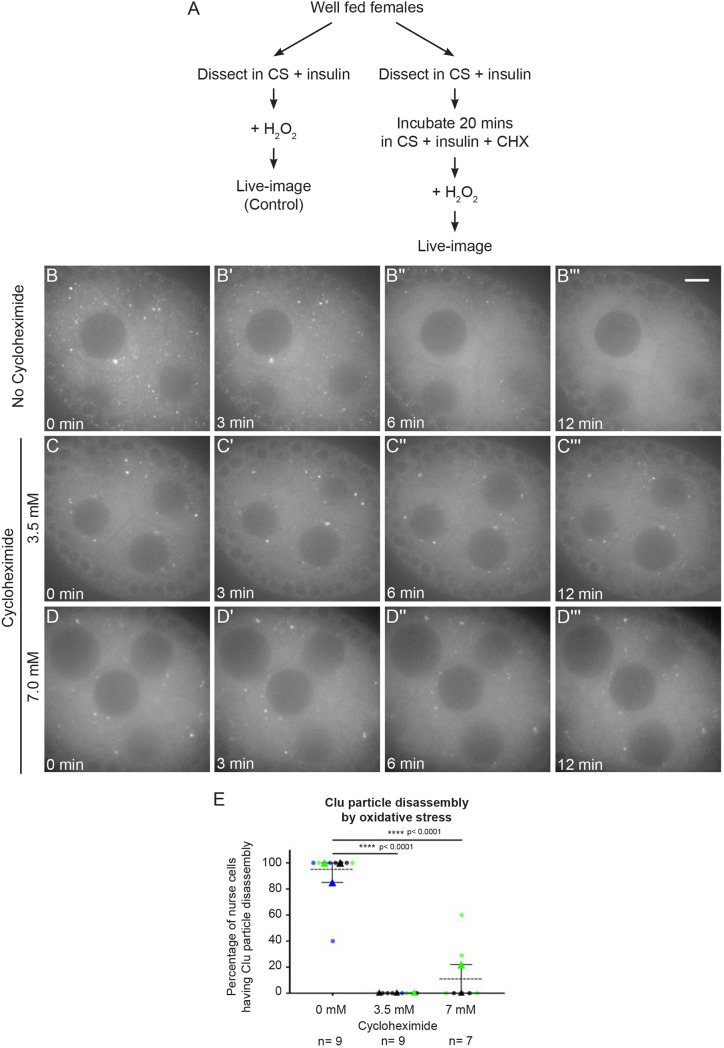
**Cycloheximide maintains Clu particles in the presence of oxidative stress.** (A) Workflow for the experiment. Ovarioles dissected from well-fed *clu^CA06604^* females were treated with CHX, then exposed to 2 mM hydrogen peroxide (C–D‴). (B–B‴) Still image of stage 7 follicle from Movie 10 showing the addition of 2 mM H_2_O_2_ leads to disassembly of Clu particles, as we have previously shown (Sheard et al., 2020). 93% of nurse cells (E, first column, *n*=9 follicles) clearly showed disassembly of Clu particles after H_2_O_2_treatment. (C–C‴) Still image of stage 7 follicle from Movie 11 and (D–D‴) Movie 12 showing CHX treatment protected Clu particles from oxidative stress-induced disassembly. None of the nurse cells pre-treated with 3.5 mM CHX for 20 min (E, second column, *n*=9/9 follicles) showed disassembly of Clu particles after H_2_O_2_ treatment, and 13% of the nurse cells pre-treated with 7 mM CHX for 20 min (E, third column, *n*=7 follicles) showed disassembly of Clu::GFP particles after H_2_O_2_ treatment. The focal plane was chosen to show at least three to four nurse cells having clear visibility of nuclear and cytoplasmic area, with ∼25% depth from the top surface of each follicle (see Materials and Methods for details). (E) The graph indicates that the effect of CHX on Clu particle maintenance is significant (****P<0.0001, one-way ANOVA followed by Tukey's post hoc test). Circles represent individual egg chamber analyzed; triangles indicate average mean of each color set. Colors represent different independent experimental group. Error bars are mean±s.e.m. *n*: the total number of follicles analyzed. Scale bar: 10 µm.

Hydrogen peroxide (H_2_O_2_) is highly toxic to cells, immediately causing a sharp increase in reactive oxygen species and oxidative damage (Vona et al., 2021). Previously, we have demonstrated that adding H_2_O_2_ to cultured ovarioles quickly disassembles Clu particles (Fig. 7B–B‴; Movie 10; Sheard et al., 2020). Given that CHX treatment of cultured ovarioles maintained Clu particles even in the absence of insulin (Fig. 6B″), we tested whether CHX treatment could maintain particles with high levels of oxidative stress (Fig. 7). We cultured ovaries from well-fed *clu^CA06604^* females, added CHX, and then exposed the ovarioles to H_2_O_2_ (Fig. 7A). Surprisingly, adding CHX maintained Clu particles in the presence of H_2_O_2_ (Fig. 7C–C‴,E; Movie 11). This was also observed for a higher CHX concentration (Fig. 7D–D‴,E; Movie 12). This observation suggests that CHX treatment and stalled ribosomes are sufficient to maintain and protect Clu particles from oxidative stress-induced particle disassembly, in addition to protection from nutritional stress.

## DISCUSSION

### Clu particle assembly requires regulated levels of functional Clu

Clu could act as a scaffold protein within particles as this is a common feature of cytoplasmic granule proteins (Buchan, 2014). We have previously demonstrated that Clu can self-associate, but we do not yet know whether Clu forms dimers or multimers or whether self-association is due to protein aggregation in Clu particles (Sen and Cox, 2016). Determining which domains are required for self-association using ectopic Clu expression has proved challenging *in vivo* given that endogenous Clu must be absent and *clu* null mutants are quite sick. Although FL-Clu rescues the *clu* null mutant, ΔDUF, ΔClu and ΔTPR do not (Fig. S1) (Sen and Cox, 2016). We have identified that the DUF, Clu and TPR domains are required for Clu to assemble in particles and for association with endogenous Clu particles, but we do not yet know whether they play a role in molecular self-association in *Drosophila*. The deletion constructs were expressed at the correct molecular masses but could be misfolded. In addition, ΔDUF and ΔClu proteins were expressed at lower levels using the ubiquitous *da*GAL4, which could explain their inability to assemble in particles, although ΔTPR was expressed at normal levels, suggesting that the missing protein domains rather than reduced protein levels are the reason. The TPR domain of CLUH has been reported to be necessary for CLUH self-association, although it appears that the self-association is not direct, suggesting CLUH forms multimers (Hémono et al., 2022a). In addition, we have previously shown that, in *Drosophila*, the TPR domain is essential for Clu–mRNA association (Sen and Cox, 2016), which was confirmed to also be the case for CLUH (Hémono et al., 2022a). Many cytoplasmic bodies and granules have associated proteins containing low-complexity or prion-like domains. The large ‘M’ domain in Clu is predicted to be intrinsically disordered, a property frequently found in RNA granule-associated proteins that is important for condensate dynamics (Lin et al., 2017). The M-domain of Clu could also be important for particle dynamics, but this has yet to be tested. Ectopically expressing FL-Clu at high levels caused Clu particles to disassemble through an unknown mechanism, which differs from what has been observation with CLUH granules (discussed below; Pla-Martin et al., 2020). Although this could be due to toxicity, that seems unlikely given that overexpression of ΔTPR and ΔDUF did not cause endogenous particle disassembly, nor did overexpression of the unrelated protein CPB. An alternative explanation could be that high concentrations of FL-Clu somehow disrupt the required stoichiometry for forming Clu particles due to the non-physiological levels. Clu levels remain the same with nutritional disruption to particles (Sheard et al., 2020). Too much functional Clu could disrupt potential liquid–liquid phase separation, which might regulate Clu particle assembly and disassembly through a gain-of-function effect caused by sequestering factors necessary for particle assembly (Alberti et al., 2019). There are few proteins known to regulate condensate disassembly; thus, a better understanding of this observation might shed light on condensate dynamics (Bard and Drummond, 2024; Brumbaugh-Reed et al., 2024).

### Assembly and maintenance of Clu particles requires translating ribosomes

As Clu associates with mRNA and ribosomal proteins, we tested the effect of translation inhibitors on assembly and disassembly with and without stress. CHX and PUR treatment are powerful tools for assessing how stalled translation affects the dynamics of particles and granules involved in the posttranscriptional regulation of mRNA. PUR decreases the amount of polysomes, terminating translation, releasing ribosomes and increasing the amount of mRNPs. In contrast, CHX prevents elongation. This results in an inhibition of polysome-to-mRNP conversion and thus increases stalled ribosomes and decreases levels of mRNPs. Stress granules are composed of mRNPs, stalled preinitiation complexes and other proteins involved in translation. Under normal conditions, low concentrations of PUR rarely leads to assembly of stress granules, but longer incubation with higher concentrations does (Kedersha et al., 2000; Bounedjah et al., 2014; Martinez et al., 2016; Ihn et al., 2024 preprint). In the presence of stress, PUR increases the number of stress granules, whereas CHX disassembles them. This dynamic is caused by an increased number of mRNPs available with PUR and a decreased number available with CHX. P-bodies generally contain proteins that are associated with mRNA decay or silencing. P-body maintenance depends on the presence of translationally repressed mRNA. In the presence of CHX, mRNA trapped in polysomes causes P-body loss. However, upon PUR treatment, the number and size of P-bodies increase due to the increase in non-translatable mRNPs (Eulalio et al., 2007; Patel et al., 2016). When treating follicles with PUR and CHX, we found that the availability of translating ribosomes regulates assembly and disassembly of Clu particles. We demonstrated this is the case with CHX feeding, which lasts for 24 h (Hwang and Cox, 2024). However, our observations for Clu particle assembly and disassembly *ex vivo* take place on the order of 7–30 min. Administering PUR *ex vivo* and *in vivo* led to disassembly of Clu particles, whereas CHX had no effect *ex vivo* and *in vivo*. This suggests that in the presence of PUR, Clu particles disassemble in response to increased mRNPs and decreased numbers of translating ribosomes. In contrast, our observations using CHX treatment suggest that stalling translation has no effect on Clu particles when they are already present. However, when particles are absent, CHX treatment blocks insulin-induced assembly *ex vivo* suggesting that increased numbers of stalled ribosomes alone cannot drive Clu particle assembly. The increased stalled ribosomes might be physically prevented from separating into Clu particles despite the unchanged Clu levels. This also suggests that insulin signaling, which initiates a strong signaling cascade, cannot overcome the CHX-mediated block in assembly.

The effect of PUR and CHX treatment on Clu particle assembly and disassembly could be due to signaling pathways induced by treatment. CHX directly stimulates TOR signaling, a potent master regulator of metabolism, by increasing the pool of amino acids due to decreased protein synthesis, but PUR treatment indirectly stimulates TOR in a context-dependent manner (Finlay et al., 2006; Iiboshi et al., 1999). Given that we have previously shown that insulin signaling is necessary and sufficient for Clu particle assembly, it is possible that particle dynamics occurs through the TOR pathway. Presently, we cannot rule this out. However, if CHX treatment upregulated TOR using our methods, we would expect that CHX treatment would drive Clu particle assembly in the absence of particles, but we did not see this (Fig. 5M′). We do not know whether our PUR and CHX treatment methods decrease global protein levels equally, although they appear to do so in cultured cell experiments (Sidhu and Omiecinski, 1998; Croons et al., 2008). The objective in this study was to observe the effect of two translational inhibitors that use different mechanisms of action on Clu particle dynamics, similar to studies of P-bodies and stress granules. The formation of stress granules occurs through the integrated stress response (ISR) through eIF2α and by TOR signaling. However, stress granule dynamics also depends on the physical characteristics of participating proteins, RNAs and mRNPs that allow condensation to occur (reviewed in Baymiller and Moon, 2023).

Nutritional and oxidative stress disassemble Clu particles. Both stressors cause decreased translation rates through ISR signaling. During nutritional stress *ex vivo* and *in vivo*, CHX treatment maintained Clu particle assembly, suggesting that, once particles are formed, stalled ribosomes present in Clu particles stabilize the particles. This was also true for oxidative damage *ex vivo* caused by H_2_O_2_. Together, these data support a model whereby *Drosophila* Clu particles harbor actively translating mRNAs, and particle assembly relies on the presence of translating ribosomes. We have yet to successfully localize ribosomes to the particles. However, data from the *Arabidopsis* Clu homolog FRIENDLY (FMT) has shown that FMT particles associate with cytoplasmic ribosomes at the mitochondrial surface (Hémono et al., 2022b). Evidence for Clu particles as sites of active translation is supported by observations of CLUH granules (Pla-Martin et al., 2020).

### Comparing *Drosophila* Clu and vertebrate CLUH subcellular localization

Clu forms large, prominent and highly dynamic particles in germ cell cytoplasm (Cox and Spradling, 2009; Sheard et al., 2020). These particles are also found in *Drosophila* somatic tissues (Sen et al., 2013; Sheard et al., 2020; Wang et al., 2015). We have previously demonstrated that Clu particles are highly sensitive to stress. Live imaging has shown that Clu particles quickly disassemble in the presence of H_2_O_2_, and for germ cells lacking particles due to starvation, adding insulin to the medium causes particle assembly in minutes (Sheard et al., 2020). Nutritional stress *in vivo* from starvation causes particle disassembly, with no decrease in protein, and particles reassemble upon feeding (Sheard et al., 2020). Various studies in vertebrate systems have demonstrated CLUH localization in the cell. In COS7 cells, CLUH exhibits a granular pattern, particularly after Triton-X 100 extraction (Gao et al., 2014). In primary hepatocytes and HeLa cells, CLUH has been reported to form granules that colocalize with some, but not all, components of stress granules, and these granules increase with stress (Pla-Martin et al., 2020). In addition, in contrast to the data shown here, CLUH overexpression induced the formation of peripheral CLUH granules in ∼40% of transfected HeLa cells (Pla-Martin et al., 2020). However, additional reports have shown CLUH is broadly diffuse in the cytoplasm in HCT116 cells, and CLUH does not localize with the stress granule component G3BP1 (Hémono et al., 2022a). In agreement with our observation, CLUH granules did not disassemble in response to CHX treatment, although these granules were the peripheral granules assembled by overexpression of CLUH (Pla-Martin et al., 2020). Finally, two groups have shown CLUH colocalizes with one or two structures composed of SPAG5 (also known as astrin), which is a mitotic spindle protein during mitosis that localizes to microtubule plus-ends in the cytoplasm during interphase (Hémono et al., 2022a; Dunsch et al., 2011; Schatton et al., 2022). It is not clear at present why Clu localization and dynamics are so different from those of vertebrate CLUH. One possibility could be that vertebrates might simply have different CLUH dynamics from *Drosophila* Clu due to differences in cell types and species. Another possibility could be that Clu particles might act differently *in vivo* compared to CLUH in cell culture experiments due to differences in cell physiology.

### Nurse cells with CHX-stabilized Clu particles have reduced cellular stress as indicated by mitochondrial localization

Starvation causes germ cell mitochondria to cluster (Sheard et al., 2020). This occurs not only with nutritional stress but appears to be a hallmark of additional cellular stressors, such as oxidative damage or stress caused by mutation in genes involved in mitochondrial function. When Clu particles are absent, mitochondria are clumped, but when the particles are present, mitochondria disperse throughout the germ cell cytoplasm in the normal pattern. This indicates that normal mitochondrial localization highly correlates with physiological levels of Clu and the presence of Clu particles (Cox and Spradling, 2009; Sheard et al., 2020; Sen et al., 2015). We found similar mitochondrial dynamics with CHX treatment. Females fed a rich CHX diet maintained assembled Clu particles and mitochondrial distribution (Fig. 5F–I″). Clu particles that remained assembled after CHX treatment followed by starvation also had normal mitochondrial localization (Fig. 6F–G″). This could be attributed to CHX increasing the level of amino acids and thus stimulating TOR activity, a downstream component of the insulin signaling pathway (Beugnet et al., 2003), although, CHX is insufficient to drive particle assembly suggesting this is not the case (Fig. 5M′). Another possibility is that, because Clu particles associate with mitochondria (Cox and Spradling, 2009; Sen et al., 2015), Clu particles assembled by CHX treatment itself could affect mitochondrial physical distribution even under stress.

In this study, we identified three protein domains of Clu that are necessary to assemble Clu particles. We also found that overexpression of functional FL-Clu causes particle disassembly. In addition, we found that Clu particles require the presence of translating ribosomes to remain assembled and that stabilizing mRNA-associated ribosomes protects particles from disassembly. Given that Clu associates with ribosomes and is a ribonucleoprotein, this supports a model whereby Clu particles are active sites of translation under non-stressed conditions but disassemble when cellular stress increases. As Clu is closely tied to mitochondrial function and binds mRNAs encoding mitochondrial proteins, particle dynamics likely affects mitochondrial function. Disassembly of particles could lead to decreased translation of the associated mRNAs, fewer mitochondrial proteins actively translated in the cytoplasm and a shift in metabolism in response to stress. There is evidence supporting this idea through studies on CLUH (Pla-Martin et al., 2020). For a better understanding of *Drosophila* Clu particles, several challenges must be overcome. There are many proteins known to associate with P-bodies and stress granules (Anderson and Kedersha, 2006; Ivanov et al., 2019). Although we and others have identified Clu/CLUH-associated proteins using co-immunoprecipitation and mass spectrometry, we have yet to localize any of them to Clu particles. In addition, we have yet to determine whether Clu particles are active sites of translation. Particles are easily seen in the nurse cells, yet using fluorescent *in situ* hybridization is challenging. Nonetheless, given the crucial role for Clu in mitochondrial function, fully understanding how these unique and novel RNP particles function will ultimately deepen our knowledge of how mitochondria respond to stress.

## MATERIALS AND METHODS

### Fly stocks

Fly stocks were maintained on standard cornmeal fly medium [Bloomington Drosophila Stock Center (BDSC), Bloomington, IN, USA, https://bdsc.indiana.edu/information/recipes/bloomfood.html]. Animals were grown at room temperature. The following stocks were used for experiments: *w^1118^*; *clu^d08713^*/*CAG*; *daGAL4*/TM6c, w*; *clu^CA06604^*/*CyO; nanosGAL4*/*TM3 Sb, w^1118^*; *UASp-cluΔDUF*::*mScarlet*/*TM3 Sb, w^1118^*; *UASp-cluΔClu*::*mScarlet*/*TM3 Sb, w^1118^*; UASp-cluΔTPR::*mScarlet*/*TM3 Sb, w^1118^*; *UASp-FLclu*::*mScarlet*/*TM3 Sb* (this study). *w^1118^*, w*; *Kr^If-1^*/*CyO*; *P{w[+mW.hs]=GAL4-da.G32}UH1* (BSC #55850), w*; *M{w[+mC]=UASp-mCherry.cpb}ZH-86Fb*/*TM3 Sb^1^* (BSC #58728), and *w^1118^*; *P{w[+mC]=GAL4*::*VP16-nanos.UTR}CG6325[MVD1]* (BSC #4937) (Van Doren et al., 1998) were obtained from the Bloomington *Drosophila* Stock Center (BDSC), Bloomington, IN, USA. *w^1118^; clu^CA06604^* (Cox and Spradling, 2009) and w*; *P{PTT-GA} tral^CA06517^* are described in (Buszczak et al., 2007). A newly eclosed fly is considered day 0.

### Transgenic flies and constructs

For the C-terminal fusion of mScarlet for live-imaging, we created a Gateway destination vector pPgateWmScarlet-i for subcloning. Briefly, the mScarlet-i coding sequence was amplified from pCytERM_mScarlet-i_N1 (Addgene #85068) using the following primers, 5′-TAGGCCACTAGTGTGAGCAAGGGCGAGGCAGT-3′and 5′-TGCTTAGGATCCTTACTTGTACAGCTCGTCCA-3′. The amplicons were subcloned into a pQUASp (Addgene #46162), placing the UASp promoter upstream of the mScarlet-i coding sequence. Ampicillin resistance was used to select positive clones, which were verified by restriction digest and sequencing. The resulting pUASp-mScarlet-i construct was converted into a Gateway destination vector using the Gateway™ Vector Conversion System (Invitrogen, cat. no. 11828029). Chloramphenicol resistance was used to select positive clones, which were verified by sequencing. The pQUASp vector was Addgene #46162 (RRID: Addgene_46162, deposited by Christopher Potter), and pCytERM_mScarlet-i_N1 vector was Addgene #85068 (RRID: Addgene_85068, deposited by Dorus Gadella; Bindels et al., 2017). Gateway entry vectors with full-length Clu (Clu_pENTR) or domain-deleted Clu (ΔDUF_pENTR, ΔClu_pENTR, and ΔTPR_pENTR) were previously described (Sen and Cox, 2016). Each entry vector was cloned into pPgateWmScarlet-i using LR Clonase mix (Invitrogen, cat. no. 11791020) following the manufacturer's directions. The resulting expression vectors were selected by ampicillin resistance and verified by sequencing. For transgenic flies, the vectors were commercially injected (BestGene Inc., Chino Hills, CA, USA).

### Live imaging for analysis of Clu particle dynamics

Live imaging with ovarioles was performed as previously described (Sheard et al., 2020) with some modifications. Ovaries were dissected in a live-imaging medium composed of Complete Schneider's (CS) medium and 200 µg/ml of insulin (insulin from bovine pancreas, Sigma-Aldrich, Burlington, MA, USA, cat. no. I6634). The CS medium was Schneider's *Drosophila* medium (Thermo Fisher Scientific, Hampton, NH, USA, cat. no. BW04351Q) supplemented with 15% heat-inactivated fetal bovine serum (CPS Serum, Parkville, MO, USA, cat. no. FBS-500HI) and penicillin (100 U/ml)-streptomycin (100 µg/ml) (Thermo Fisher Scientific, cat. no. BW17602E). After separating ovarioles from an ovary and eliminating the connected muscle sheath and nerve fibers, the tissues were transferred into a 35 mm MatTek glass bottom dish (MatTek Corporation, Ashland, MA, USA, cat. no. P35G-0-20-C) with a live-imaging medium. The follicles were mainly chosen between stages 5 to 7, previtellogenic stages, for better observation with nurse cells. The focal plane was selected to have at least three to four nurse cells with a clear visibility of nuclear and cytoplasmic areas, with ∼25% depth from the top surface of a follicle. Follicle stages were determined by follicle size as described previously (Spradling, 1993). Live images were obtained using a Nikon A1 plus Piezo Z Drive Confocal microscope with a 60× objective lens (Nikon Corporation, Tokyo, Japan) or a Nikon Eclipse Ti2 spinning disk microscope with a 100× objective lens (Nikon Corporation, Tokyo, Japan). Fiji ImageJ was utilized to analyze confocal images (Schindelin et al., 2012).

### Detection and analysis of Clu particle assembly

Fiji Image J (Schindelin et al., 2012) was utilized to detect and count the Clu particles co-labeled with endogenous Clu::GFP and ectopic Clu::mScarlet. Briefly, a line was applied to a single-frame image, and a multi-channel plot histogram was generated with the tools, Plot Profile and Visualization_toolset. After getting the image by Difference of Gaussian for each fluorescence channel, the tool DiAna was used to analyze Clu particles co-labeled with endogenous and ectopic Clu proteins (Gilles et al., 2017).

### Live imaging for Clu particle dynamics after puromycin, cycloheximide and hydrogen peroxide treatment

The working solution for each chemical was prepared just before performing an experiment as follows: 10 mg/ml puromycin (PUR, Gibco™, Waltham, MA, USA, cat. no. A1113803) was diluted to 20 µM in a medium composed of CS medium and 100 µg/ml of insulin (CS/Ins); cycloheximide powder (CHX, Sigma-Aldrich, Burlington, MA, USA, cat. no. C7698) was dissolved in CS for 14 mM stock solution, which was further diluted to 3.5 mM and 7 mM CHX-containing CS or CS/Ins media; and 30% H_2_O_2_ (Sigma-Aldrich, Burlington, MA, USA, cat. no. H1009) was diluted to 4% H_2_O_2_ in CS/Ins or CHX-containing CS/Ins. Ovaries were dissected as described above with a corresponding medium, CS or CS/Ins, depending on the purpose of each experiment. To test the PUR effect on Clu particle dynamics, dissected ovaries with CS/Ins medium were transferred into a 35 mm MatTek glass bottom dish with 50 µl of CS/Ins medium, and live imaging was performed after adding 50 µl of CS/Ins containing 20 µM puromycin to the ovaries to make a final concentration of 10 µM puromycin. To test the CHX effect on Clu particle dispersion, dissected ovaries with CS/Ins medium were incubated for 20 min with 3.5 mM CHX-containing CS/Ins (CS/Ins/CHX) following two washes with the same medium, and then a live-imaging was performed. To test the CHX effect on Clu particle formation, ovaries were dissected from starved animals in CS, incubated for 20 min with 3.5 mM CHX-containing CS (CS/CHX) following two washes with the same medium, re-washed twice with CS/CHX containing 100 µg/ml insulin (CS/CHX/Ins), and then switched to CS/CHX/Ins for immediate live imaging. To test the CHX effect on particle dispersion without insulin, ovaries were dissected from *w^1118^*; *clu^CA06604^* in CS, washed twice with CS containing 3.5 mM CHX (CS/CHX), incubated for 30 min with the same medium and then live images were obtained. To produce oxidative stress, dissected ovaries were incubated with 50 µl of CS/Ins or CS/CHX/Ins for 20 min following two washes with a corresponding medium. Live imaging was performed after adding 50 µl of each corresponding medium containing 4 mM H_2_O_2_ to make a final concentration of 2 mM H_2_O_2_. Selection of follicle stages and a focal plane was performed as described above. Images were obtained using a Nikon A1plus Piezo Z Drive confocal microscope with a 60× objective lens or a Nikon Eclipse Ti2 spinning disk microscope with a 100× objective lens.

### Preparation of puromycin and cycloheximide solution for wet yeast paste to feed flies

PUR and CHX were dissolved in water to prepare a 2 mM stock solution and 10 mM stock solution, respectively. The stock solutions were aliquoted and stored at −20°C until use. The desired concentration of PUR or CHX was prepared by serial dilution of the stock solution. 0.3 g of active dry yeast powder (Red Star^®^ Yeast) was mixed with 450 µl of inhibitor solution to create a yeast paste, which was provided daily as needed.

### Puromycin and cycloheximide feeding for ovary analysis

Ten female day 0 adult flies and ten male day 0 adult flies were collected in a standard food vial with wet yeast paste, and on day 3, female adults were separated from males. The food vial with fresh wet yeast paste was switched every day to make flies fatten until day 4. On day 4, all female flies in each vial were transferred to a standard food vial containing freshly made yeast paste with PUR or CHX. At 24 h after feeding yeast paste containing each translation inhibitor, fly ovaries were dissected in Grace's insect medium (Invitrogen, cat. no. 1595030). To determine the effect of starvation after CHX feeding, after feeding CHX for 24 h, the flies were transferred to an empty vial with a wet piece of Kimwipe with water and maintained for 5 h. The flies were then dissected and immunostained.

### Immunostaining

Ovaries were dissected with Grace's insect medium and fixed for 20 min (4% paraformaldehyde and 20 mM formic acid in Grace's insect medium). After washing with antibody wash solution (AWS; 0.1% Triton X-100 and 1% BSA in phosphate-buffered saline) three times for 20 min, the tissue was stained with primary antibody overnight at 4°C. After washing with AWS three times for 20 min, the tissues were stained with secondary antibodies overnight at 4°C, then washed with AWS twice for 20 min and stained with 5 ng/ml DAPI solution for 10 min. After removing the DAPI solution, the tissues were mounted in Vectashield Antifade Mounting Medium (Vector Laboratories, Newark, CA, USA, cat. no. H-1000). Images were obtained using a Zeiss LSM 980 confocal laser scanning microscope (Carl Zeiss Microscopy LLC, White Plains, NY, USA). The follicles were mainly chosen between stages 5 to 7, the previtellogenic stages, for better observation in nurse cells. The focal plane was selected to ensure at least three to four nuclei were clearly visible in a nurse cells, with ∼25% depth from the top surface of a follicle but also to avoid a dim fluorescence signal due to a deeper depth of imaging. The numbers of dissected and observed follicles and replicates are described in a graph within each figure. The following antibodies were used: guinea pig anti-Clu N-terminus (1:2000; Cox and Spradling, 2009), rat anti-mScarlet-i sdAb-FluoTag-X2 (1:1000, NanoTag Biotechnologies, Goettingen, Germany, cat. no. N1302-At488-L, RRID:AB_3075967), chicken anti-mCherry (1:1000, Novus Biologicals, Centennial, CO, USA, cat. no. NBP2-25158 RRID:AB_2636881), mouse anti-ATP5A1 (1:1000, Abcam, Cambridge, UK, cat. no. 14748, RRID:AB_301447), or Thermo Fisher Scientific, Waltham, MA, USA, cat. no. 439800, RRID:AB_2533548), goat anti-guinea pig IgG conjugated to Alexa Fluor 488 (1:1000, Molecular Probes, Waltham, MA, USA, cat. no. A11073, RRID:AB_2534117), goat anti-guinea pig IgG conjugated to Alexa Fluor 633 (1:1000, Thermo Fisher Scientific, Waltham, MA, USA, cat. no. A21105, RRID:AB_2535757), goat anti-chicken IgY conjugated to Alexa Fluor 568 (1:500, Thermo Fisher Scientific, Waltham, MA, USA, cat. no. A11041, RRID:AB_2534098), goat anti-mouse IgG2b conjugated to Alexa Fluor 488 (1: 500, Thermo Fisher Scientific, Waltham, MA, USA, cat. no. A21141, RRID:AB_2535778), goat anti-mouse IgG2b conjugated to Alexa Fluor 568 (1: 500, Thermo Fisher Scientific, Waltham, MA, USA, cat. no. A21144 RRID:AB_2535780).

### *clu* mutant rescue

Flies overexpressing Clu in the *clu* null background were generated by crossing *w^1118^*; *clu^d08713^*/*CAG*; d*aGAL4*/*TM6c* virgins with *w^1118^*; *clu^d08713^*/*CAG*; *UASp-FLclu*::*mScarlet*/*TM3 Sb* males. From this cross, ten virgin females of *w^1118^*; *clu^d08713^*/*clu^d08713^*; *daGAL4*/*UASp-FLclu*::*mScarlet* were crossed with ten *w^1118^* males in a standard food vial, then removed after 3 days. The vials were kept at room temperature for an additional 5 days and then imaged. Sibling females, *w^1118^*; *clu^d08713^*/*CAG*; *daGAL4/TM3b* and *w^1118^*; *clu^d08713^*/*CAG*; *daGAL4*/*UASp-FLclu*::*mScarlet*, were used for control.

### Western blot analysis

Ten ovary pairs were dissected from day 5–7 well-fed adult females and homogenized with a whole-cell lysis buffer composed of 50 mM Tris-HCl pH 8.0, 150 mM sodium chloride, 1 mM EDTA, 1% NP-40, 0.1% sodium dodecyl sulfate and Complete-mini EDTA-free protease inhibitor (Roche Applied Science, Indianapolis, IN, USA, cat. no. 11836170001) by 30 times of strokes with a pestle. Insoluble material was removed by centrifugation (12,000 ***g*** for 20 mins at 4°C), and the subsequent supernatant was collected as a fraction of whole cellular proteins. After determining protein concentration using a Pierce BCA Protein Assay Kit (Thermo Fisher Scientific, Waltham, MA, USA, cat. no. 23227), 40 µg of each sample was separated by SurePAGE™, Bis-Tris gel (GensScript, Piscataway, NJ, cat. no. M00653) and transferred to a nitrocellulosemembrane. The nitrocellulose membrane was probed with guinea pig anti-CluN antibody (1:10000, Cox and Spradling, 2009), chicken anti-mCherry antibody (1:1000, Novus Biologicals, Centennial, CO, USA, cat. no. NBP2-25158, RRID:AB_2636881), and mouse anti-tubulin antibody (1:5000, Developmental Studies Hybridoma Bank, University of Iowa, Iowa City, IA, cat. no. AA4.3). Western blots were performed in triplicate and images were analyzed using Fiji ImageJ (Schindelin et al., 2012). To analyze the expression levels of ectopic proteins, mScarlet detected by anti-mCherry antibody was normalized with tubulin. The data was analyzed using GraphPad Prism, as described below. See Fig. S5 for uncropped images of blots presented in this study.

### Live-imaging for P-bodies

To test the CHX effect on P-body disassembly, ovaries were dissected from w*; *P{PTT-GA} tral^CA06517^* flies in CS medium, and then incubated for 30 min with CS containing 3.5 mM CHX (CS/CHX) following two washes with the same medium. Live images were obtained using a Nikon Eclipse Ti2 spinning disk microscope with a 100× objective lens (Nikon Corporation, Tokyo, Japan). The follicles were mainly chosen in stages 7–8 that show greater numbers of P-bodies compared to earlier stages. The focal plane was selected to have at least three to four nurse cells with a clear visibility of nuclear and cytoplasmic area, with ∼25% depth from the top surface of a follicle. Changes in P-bodies were determined as a subjective measurement by a researcher who was aware of the experimental conditions.

### Graphs and statistics

The graphs and statistical analysis by the unpaired two-tailed *t*-test and one-way ANOVA test with multiple comparisons followed by Tukey's post hoc test were generated using GraphPad Prism (GraphPad Software, Boston, Massachusetts USA, www.graphpad.com).

## Supplementary Material

10.1242/joces.263730_sup1 Supplementary information
