# Supplementary material for: Drosophila Clu ribonucleoprotein particle dynamics rely on the availability of functional Clu and translating ribosomes
Source: J Cell Sci. 2025 May 9;138(9):jcs263730. doi: 10.1242/jcs.263730 (PMC12136168; doi:10.1242/jcs.263730)
Supplement: Supplementary information [file joces-138-263730-s1.pdf]

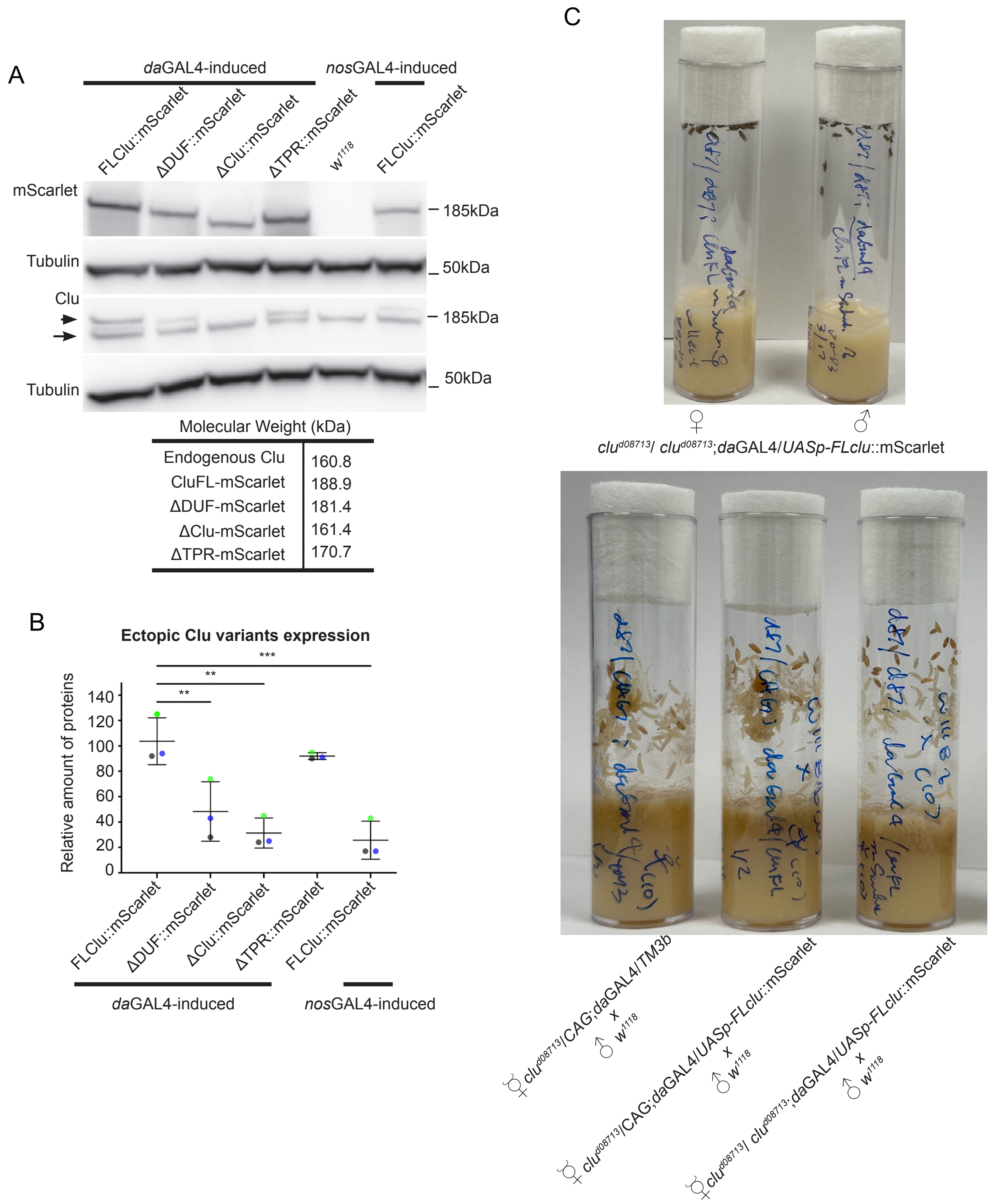

Fig. S1. Ectopic Clu expression rescues *clu* mutants. (A) Representative western blots of the ovaries from the females expressing ectopic Clu. Quantification of each Clu variant expression was normalized to tubulin expression. The arrow indicates endogenous Clu. The arrow head indicates mScarlet-tagged Clu variants. Table indicates the molecular weight of each variant protein. (B) Relative expression levels of ectopic Clu variants. Expression of *da*GAL4-induced FLClu (first column, arithmetic mean: 104) is a four-fold increase than an expression of *nanos (nos)*GAL4-induced FLClu (fifth column, arithmetic mean: 26). w^{1118} is a negative control of ectopic expression. Unpaired two-tailed t-test was performed. **: p<0.01, ***: p<0.001. (C) *da*GAL4-induced ectopic FLClu::mScarlet rescued the sterile, short-lived *clu* null mutant phenotype. Top pannel: Females (vial on the left) and males (vial on the right) overexpressing *da*GAL4-induced ectopic FLClu::mScarlet in *clu* null mutants were collected and maintained for two weeks. Bottom panel: Eggs laid by the females overexpressing of *da*GAL4-induced ectopic FLClu::mScarlet were hatched and grown (vial on the right, clu^{d08713}/clu^{d08713};*daGAL4*/*UASp-FLclu*::mScarlet x w^{1118}). Sibling control females (from left: clu^{d08713}/*CAG*;*daGAL4*/*TM3b*, clu^{d08713}/*CAG*;*daGAL4*/*UASp-FLclu*::mScarlet) were crossed with w^{1118} males and their eggs were hatched and grown.

Journal of Cell Science • Supplementary information

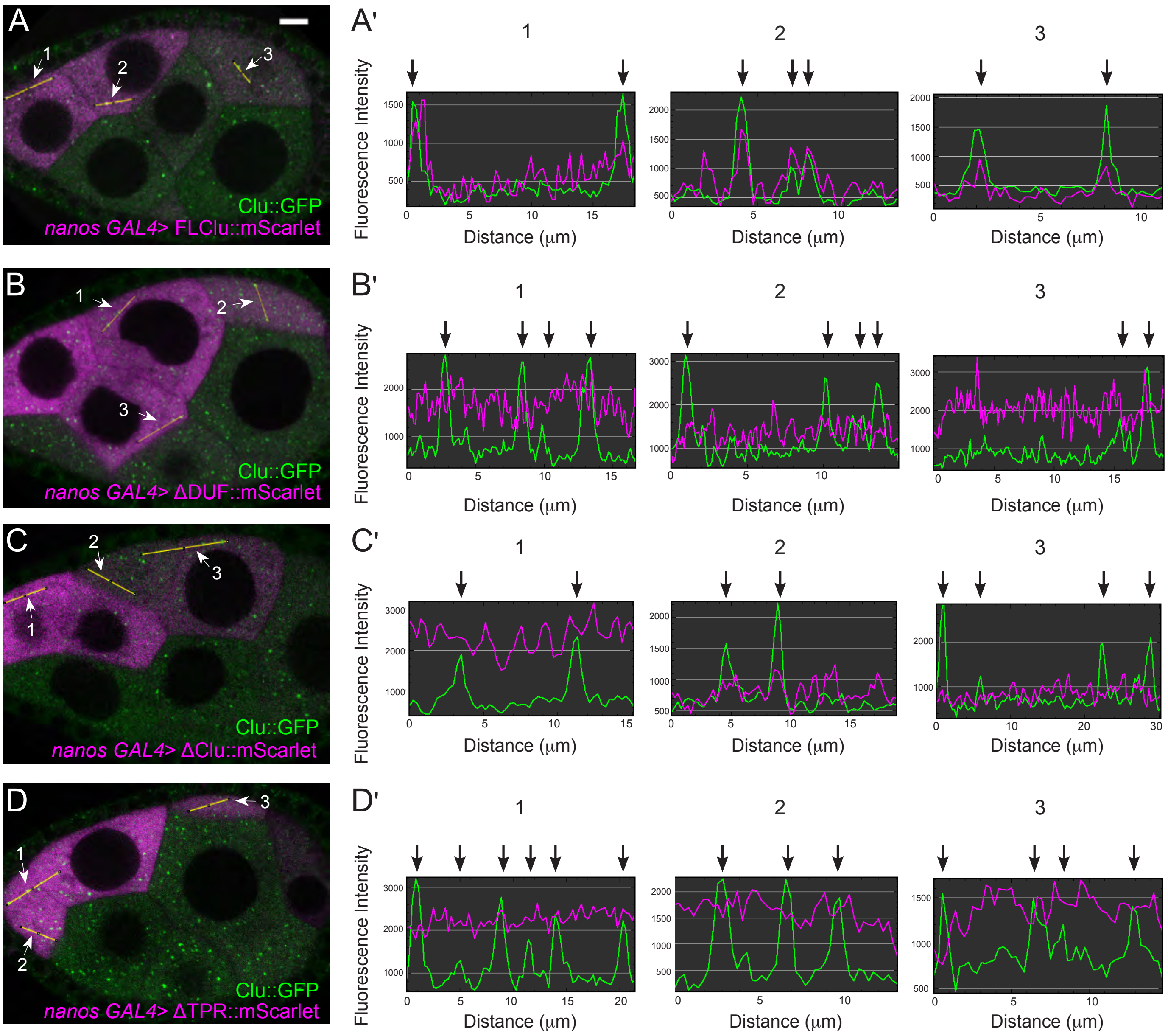

Fig. S2. DUF, Clu, and TPR domains are required for Clu particle association. (A) The same still-image in Fig. 2B” of a follicle from a *clu*^{CA06604} /+; *nanos* (*nos*) *GAL4*/*UASp-FLclu*::mScarlet female. (A’) Multi-channel histograms generated with three lines applied in A. A relatively high level of each fluorescence peak compared to the baseline indicates a single Clu::GFP particle (green) or a single Clu::mScarlet particle (magenta). The particles colocalized were quantified in Fig. 2G. (B,C,D) The same still-image in Fig. 2C”,D”,E” of follicles ectopically expressing each domain deletion construct, repectively. (B’,C’,D’) Multi-channel histograms generated with three lines in each corresponding image. (A’,B’,C’,D’) Each arrow indicates a single Clu::GFP particle (green). (A-D’, merge) Green = Clu::GFP, magenta = mScarlet. Scale bar: 10 μm in A for A-D.

Journal of Cell Science • Supplementary information

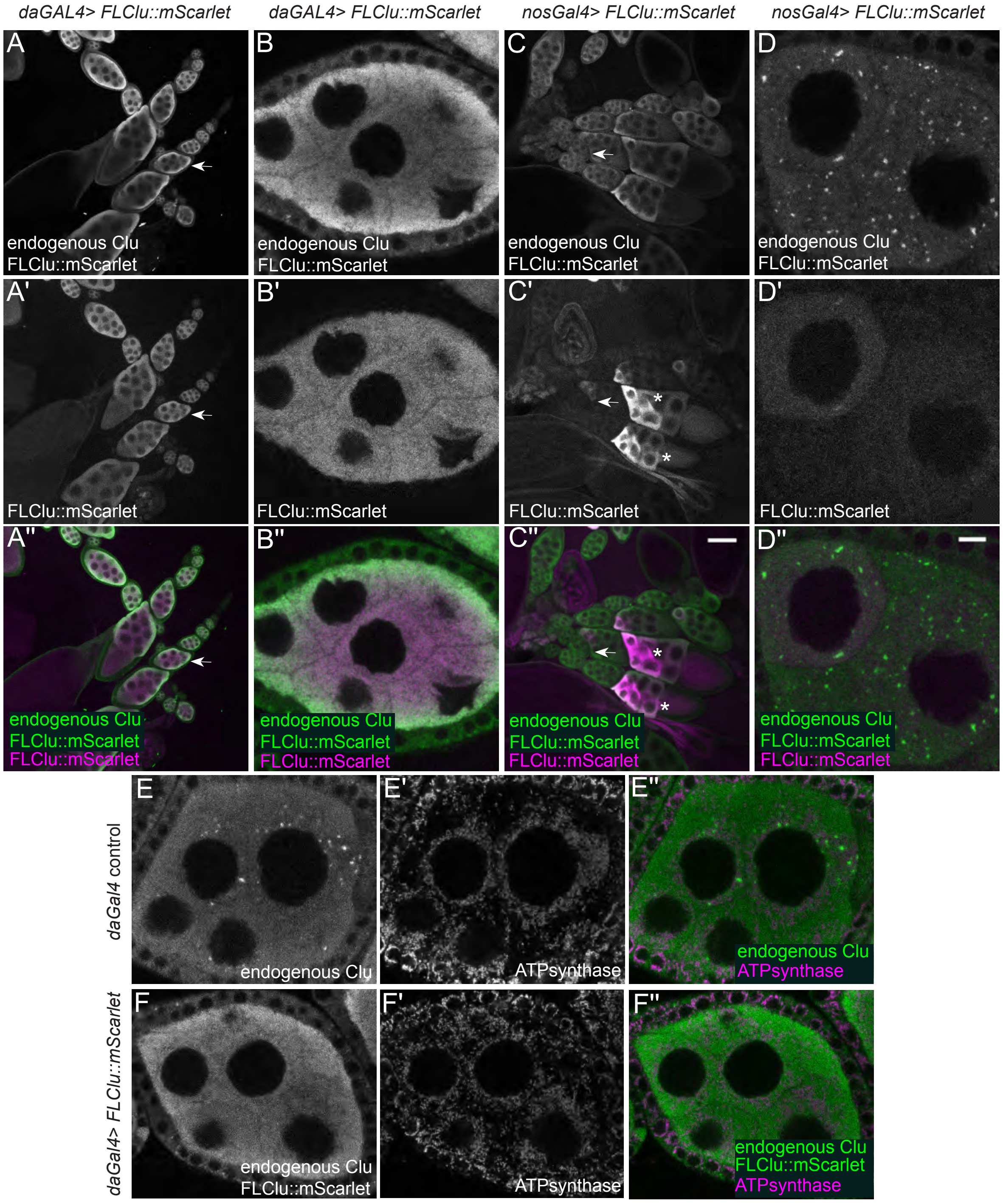

Fig. S3. *da*GAL4 induces a high level of ectopic Clu expression. (A-A”) Immunostaining of dissected ovaries and (B-B”) a representative stage 7 egg chamber (indicated by an arrow in A-A”) from a female overexpressing *da*GAL4-induced FLClu::mScarlet. (C-C”) Immunostaining of dissected ovaries and (D-D”) a representative stage 7 egg chamber (indicated by an arrow in C-C”) from a female overexpressing *nanos* (*nos*) GAL4-induced FLClu::mScarlet. (A-A”,C-C”) Dissected ovarioles were imaged with a 18 µm thickness of z-stacks with an interval of 1.8 µm. (B-B”,D-D”) Stage 7 egg chamber follicles were imaged with a 1.2 µm thickness of z-stacks with an interval of 0.42 µm. The focal plane was selected by ensuring at least three to four nuclei were clearly visible in nurse cells, but also to avoid dim fluorescence signals due to deeper depth. (A’,B’) daGAL4 induced high expression of ectopic FLClu::mScarlet in germ cells and very low expression in somatic follicle cells. (C’,D’) *nos*GAL4 induced very low levels of clonal expression of ectopic FLClu::mScarlet in stages younger than Stage 9 compared to *da*GAL4. Expression dramatically increased at Stage 9 (C’,C”, asterisks). Laser power for the mScarlet labeling in D’-D” was increased to visualize the staining, but the gain remained the same (LaserPower: 0.7% (B’,B”, D’,D”, anti-Clu), 2.2% (B’,B”, anti-mScarlet), 6.6% (D’,D”, anti-mScarlet)). (E-F”) Immunostaining of egg chamber from a female overexpressing *da*GAL4 -induced FLClu::mScarlet (F-F”) and its sibling female without ectopic expression (E-E”) both show normal mitochondrial distribution (E’,F’). Images were obtained using a Zeiss LSM 980 confocal laser scanning microscope (Carl Zeiss Microscopy LLC, White Plains, NY, USA). (A,B,C,D,E,F) White = anti-Clu. (A’,B’,C’,D’) White = anti-mScarlet. (E’,F’) White = anti-ATPsynthase. (A”,B”,C”,D”, merge) Green = anti-Clu, magenta = anti-mScarlet. (E”,F”, merge) Green = anti-Clu, magenta = anti-ATPsynthase. For A,A”,B,B”,C,C”,D,D”,E,E”,F,F”: Note: anti-Clu antibody also recognizes the mScarlet transgene. Scale bar: 100 µm in C” for A-A”,C-C”. Scale bar: 10 µm in D” for B-B”,D-F”.

Journal of Cell Science • Supplementary information

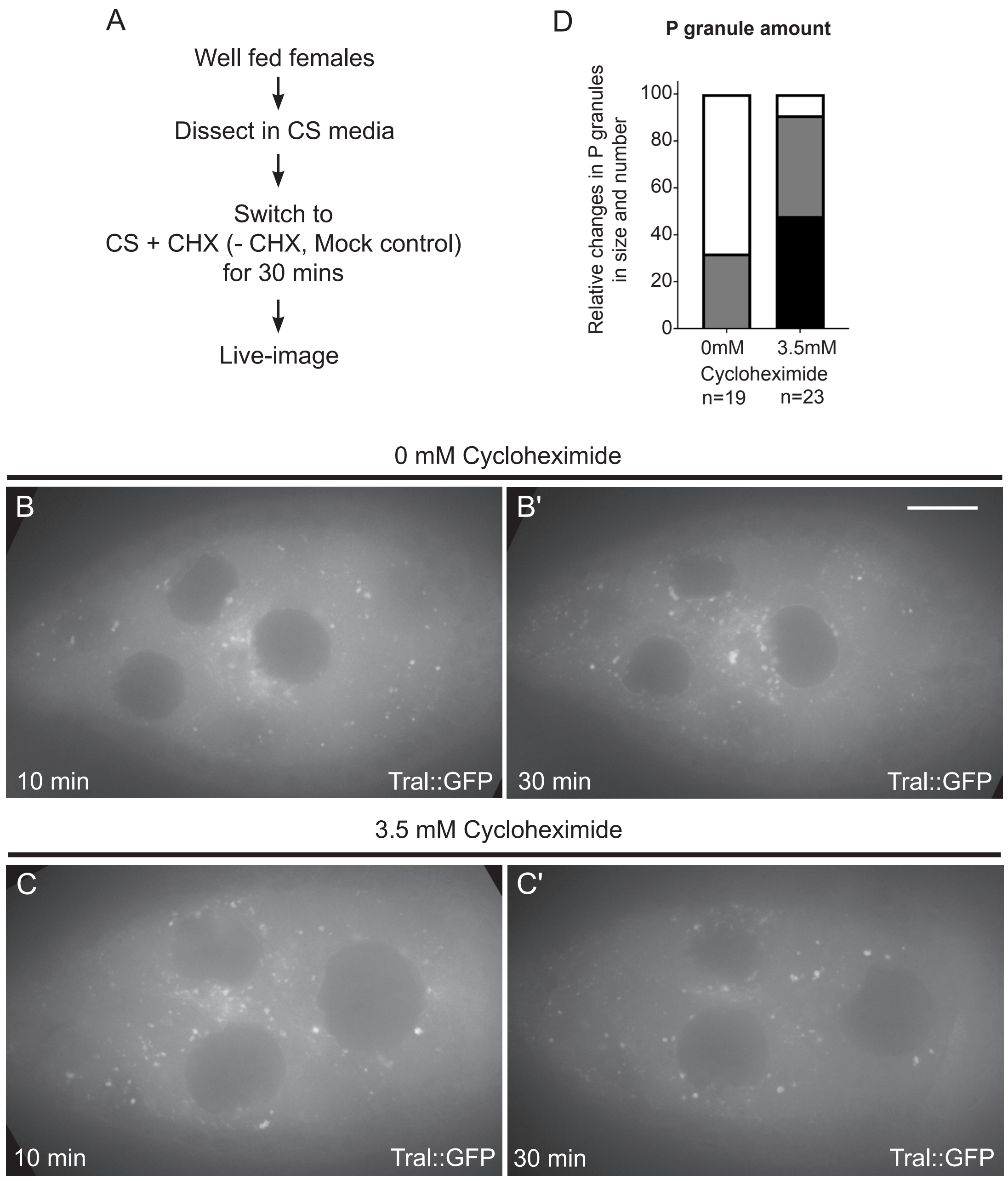

Fig. S4. Cycloheximide causes reduced sizes and numbers of P-bodies ex vivo. (A) Workflow for the experiment. Well-fed *tral*^{CA06517} females were treated with 3.5 mM CHX for 30 minutes, and then live-imaged (B-C'). (B-B') Still-image of stage 8 follicle showing a mock-control increased P-bodies. 68% of nurse cells had increased numbers and sizes of P-bodies after 30 minutes without insulin and CHX, and 32% of nurse cells had no changes (D, first column, gray, n=19 follicles). (C-C') Still-image of stage 8 follicle showing 3.5 mM CHX treatment decreases P-bodies. 48% of nurse cells had decreased numbers and sizes of P-bodies after 30 minutes without insulin but with 3.5 mM CHX treatment (D, second column, black), 43% of nurse cells had no changes (D, second column, gray), and 9% of nurse cells had increased (D, second column, white, n=23 follicles). Images are 2 µm projections assembled from 0.5 µm sections. The focal plane was selected to have at least three to four nurse cells with a clear visibility of nuclear and cytoplasmic area, aiming for ~ 25% depth from the top surface of a folli-cle. Changes in P-bodies were determined by subjective measurement. (D) Black=decreased, gray=no change, white=increased. Scale bar: 20 µm in B' for B-C'.

Journal of Cell Science • Supplementary information

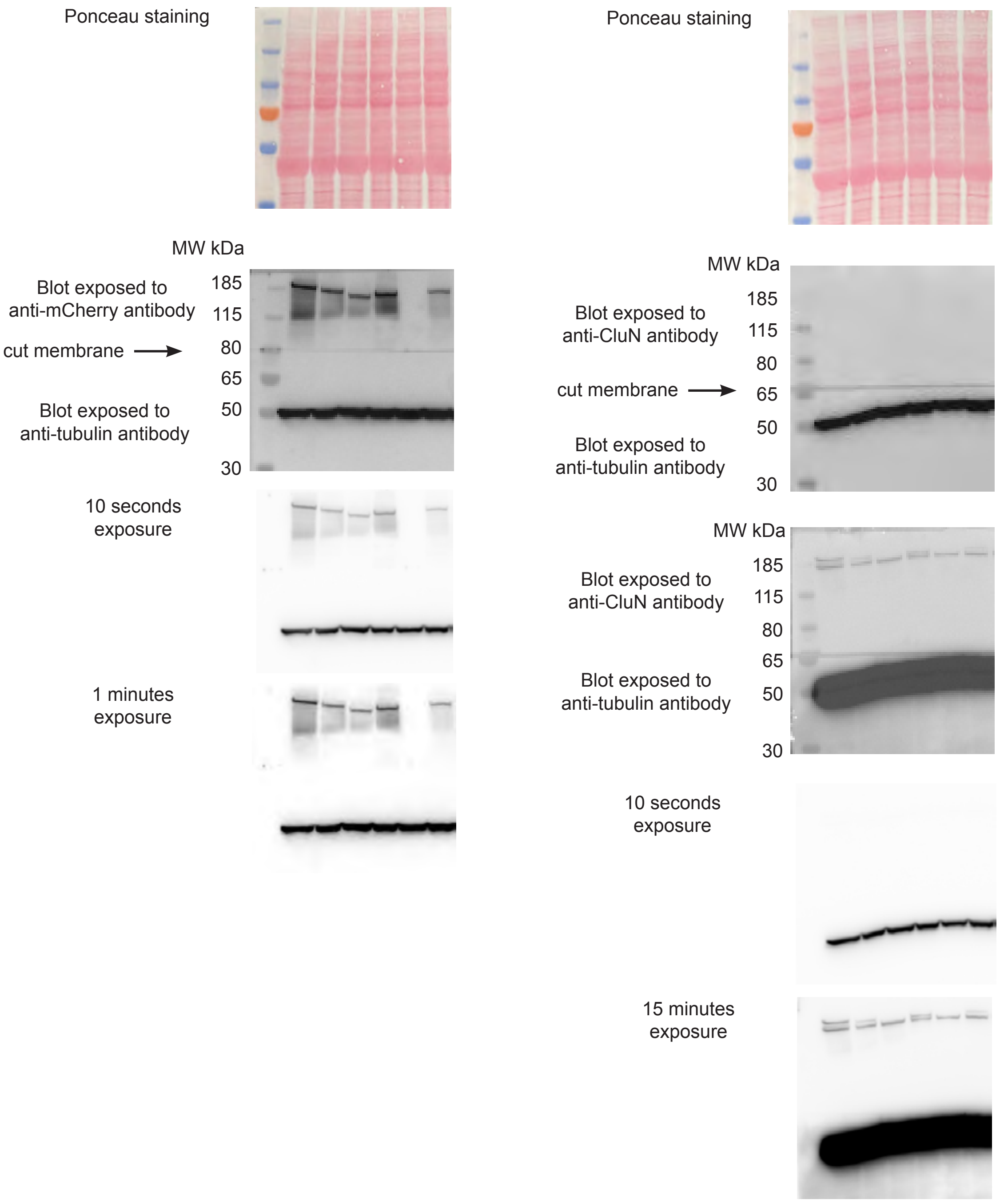

Fig. S5. Uncropped Western blots and Ponceau staining.

Journal of Cell Science • Supplementary information

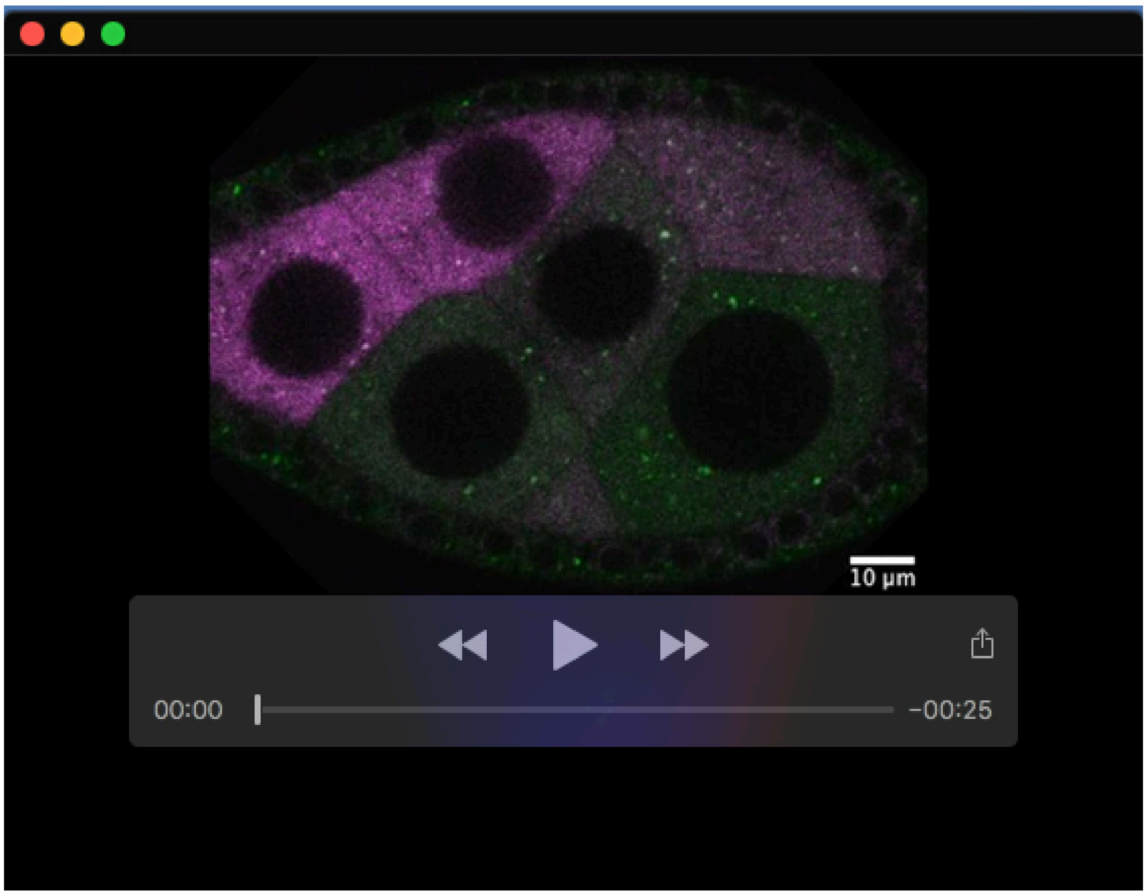

Movie 1. Live-imaging of the follicles from *clu*^{CA06604}/+; *nos GAL4*/*UASp-FLClu*::*mScarlet* female. Endogenous Clu GFPTrap (green) and ectopic FLClu::mScarlet (magenta) were recorded at 11-second intervals for 5 minutes using a Nikon A1 confocal laser scanning microscope. Video was recorded at ten frames per second.

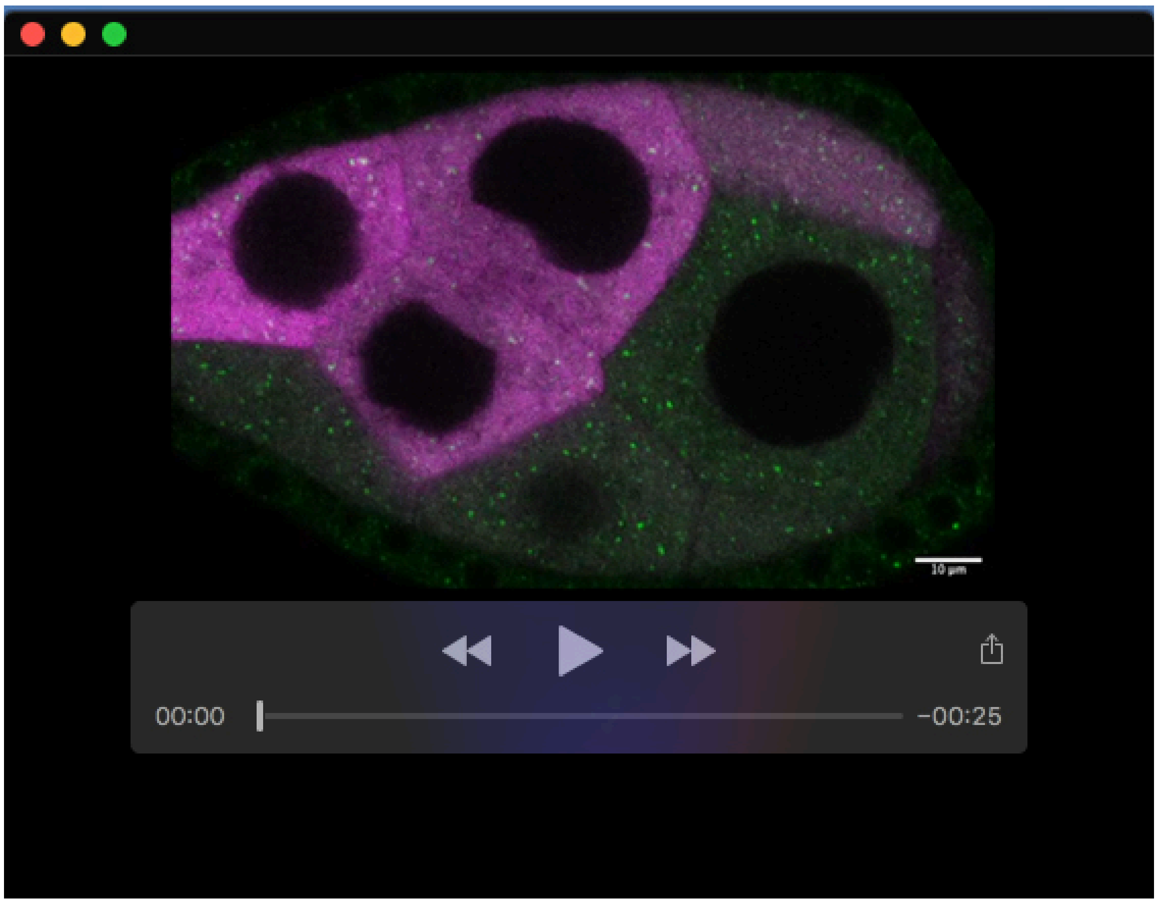

Movie 2. Live-imaging of the follicles from *clu*^{CA06604}/+; *nos GAL4*/*UASp-clu⊿DUF::mScarlet* female. Endogenous Clu GFPTrap (green) and ectopic ⊿DUF::mScarlet (magenta) were recorded at 4.2-seccond intervals for 2 minutes using a Nikon A1 plus confocal microscope. Video was recorded at ten frames per second.

Journal of Cell Science • Supplementary information

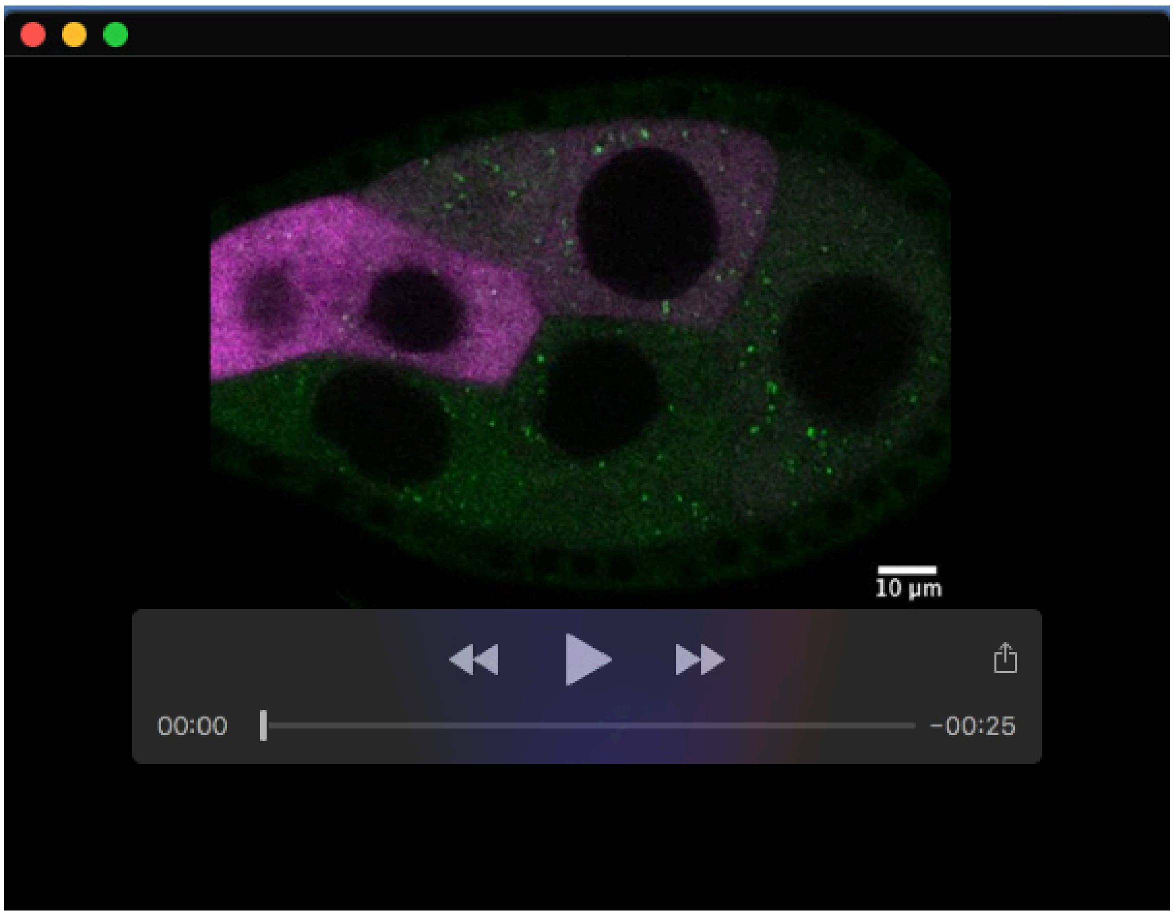

Movie 3. Live-imaging of the follicles from ***clu***^{CA06604}**/+;** ***nos GAL4/UASp-cluΔClu::mScarlet*** **female.** Endogenous Clu GFPTrap (green) and ectopic ΔClu::mScarlet (magenta) were recorded at 2.1-second intervals for 2 minutes using a Nikon A1 plus confocal microscope. Video was recorded at ten frames per second.

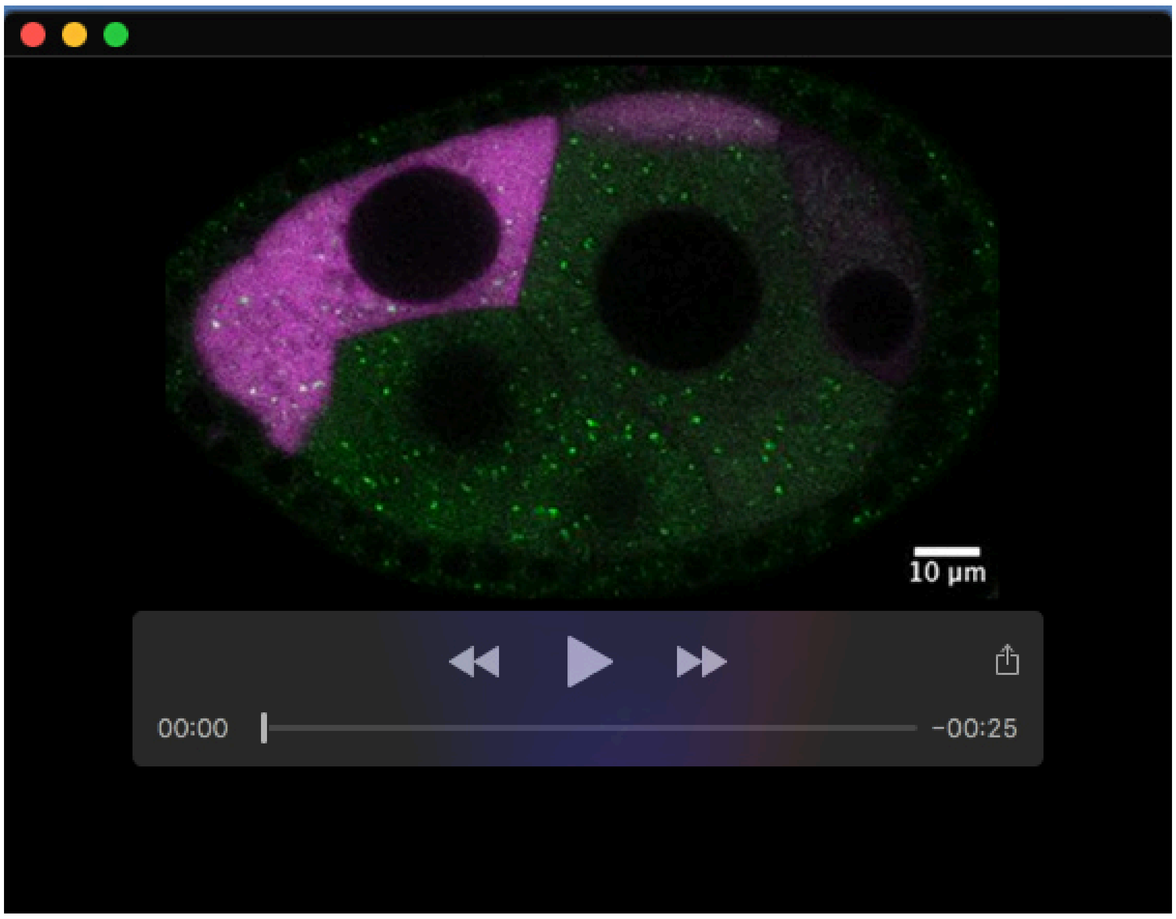

Movie 4. Live-imaging of the follicles from ***clu***^{CA06604}**/+;** ***nos GAL4/UASp-cluΔTPR::mScarlet*** **female.** Endogenous Clu GFPTrap (green) and ectopic ΔTPR::mScarlet (magenta) were recorded at 2.1-second intervals for 2 minutes using a Nikon A1 plus confocal microscope. Video was recorded at ten frames per second.

Journal of Cell Science • Supplementary information

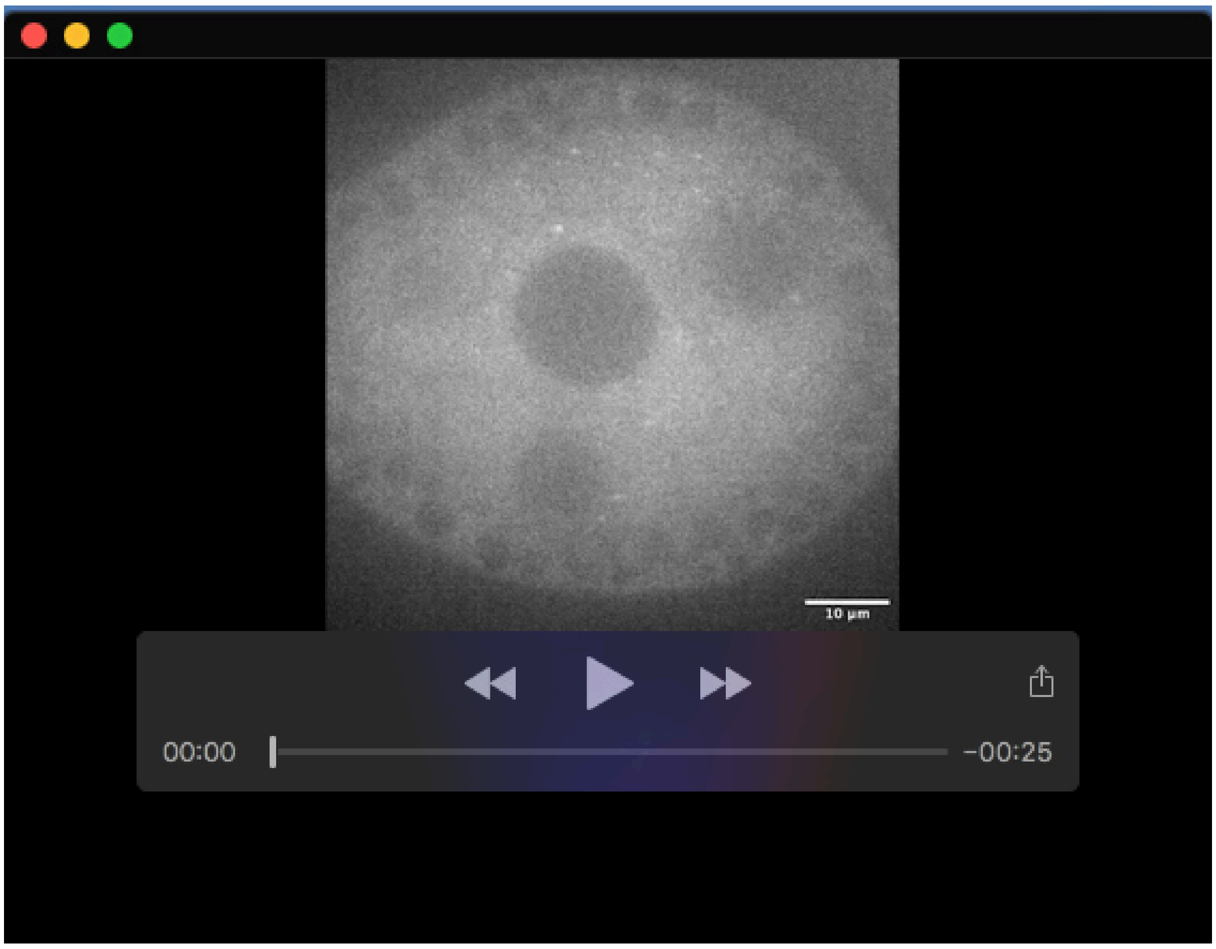

Movie 5. Live-imaging of the follicles from *clu*^{CA06604} female exposed to puromycin. Dissected ovarioles were exposed to 10 μM puromycin and recorded at 20-second intervals for 10 minutes using a Nikon Eclipse Ti2 spinning disk microscope. Video was recorded at ten frames per second.

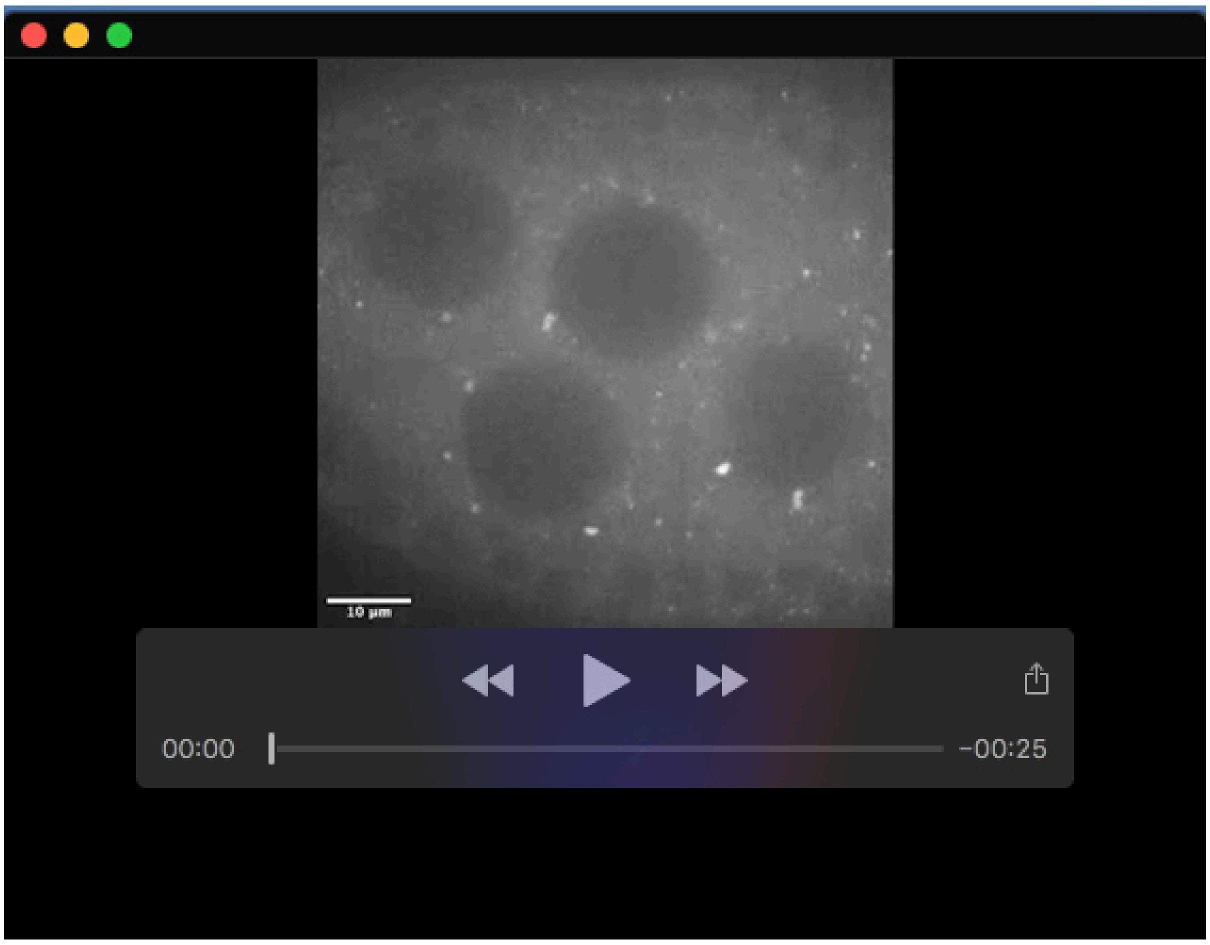

Movie 6. Live-imaging of the follicles from well-fed *clu*^{CA06604} female. The object was recorded at 0.2-second intervals for 3 minutes using a Nikon Eclipse Ti2 spinning disk microscope. Video was recorded at 240 frames per second.

Journal of Cell Science • Supplementary information

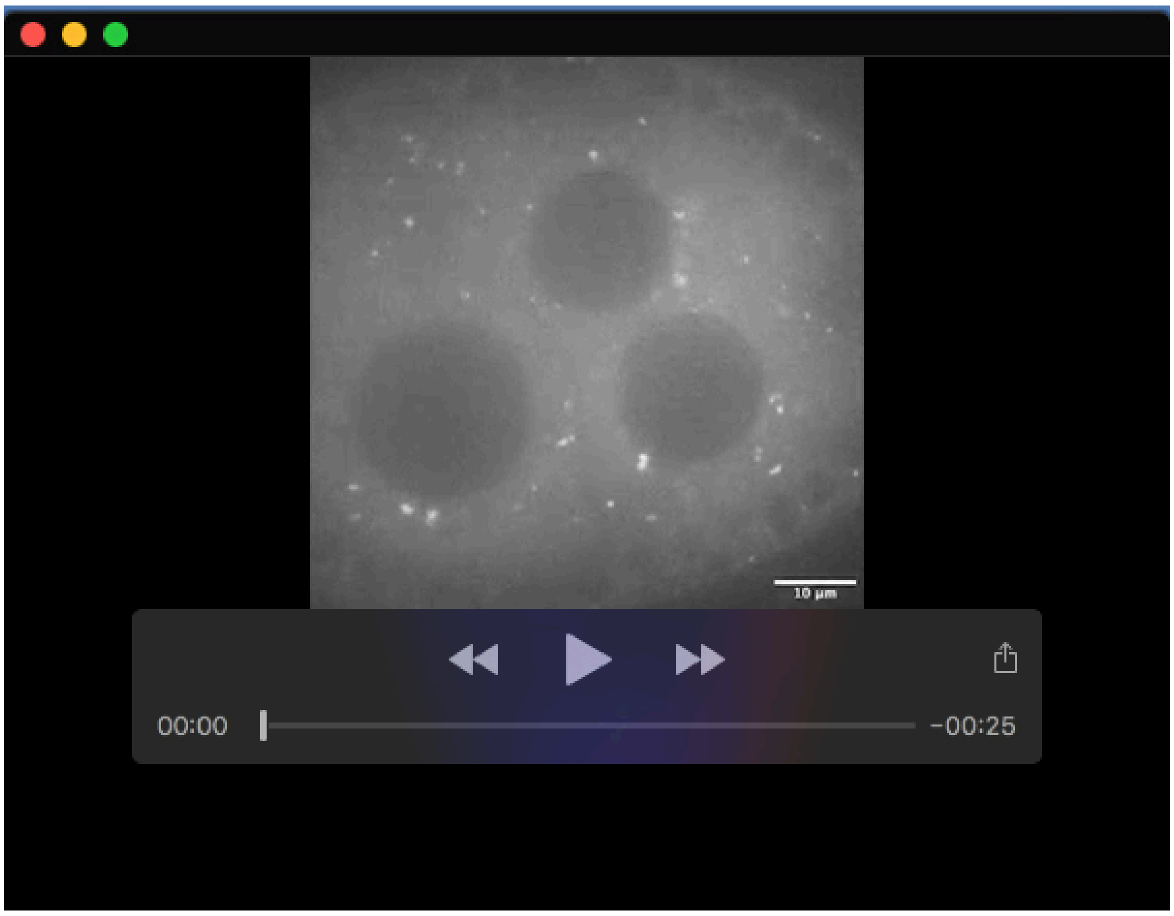

Movie 7. Live-imaging of the follicles from well-fed *clu*^{CA06604} female exposed to cycloheximide. Dissected ovarioles were exposed to 3.5 mM cycloheximide for 20 minutes and recorded at 0.2-second intervals for 3 minutes using a Nikon Eclipse Ti2 spinning disk microscope. Video was recorded at 240 frames per second.

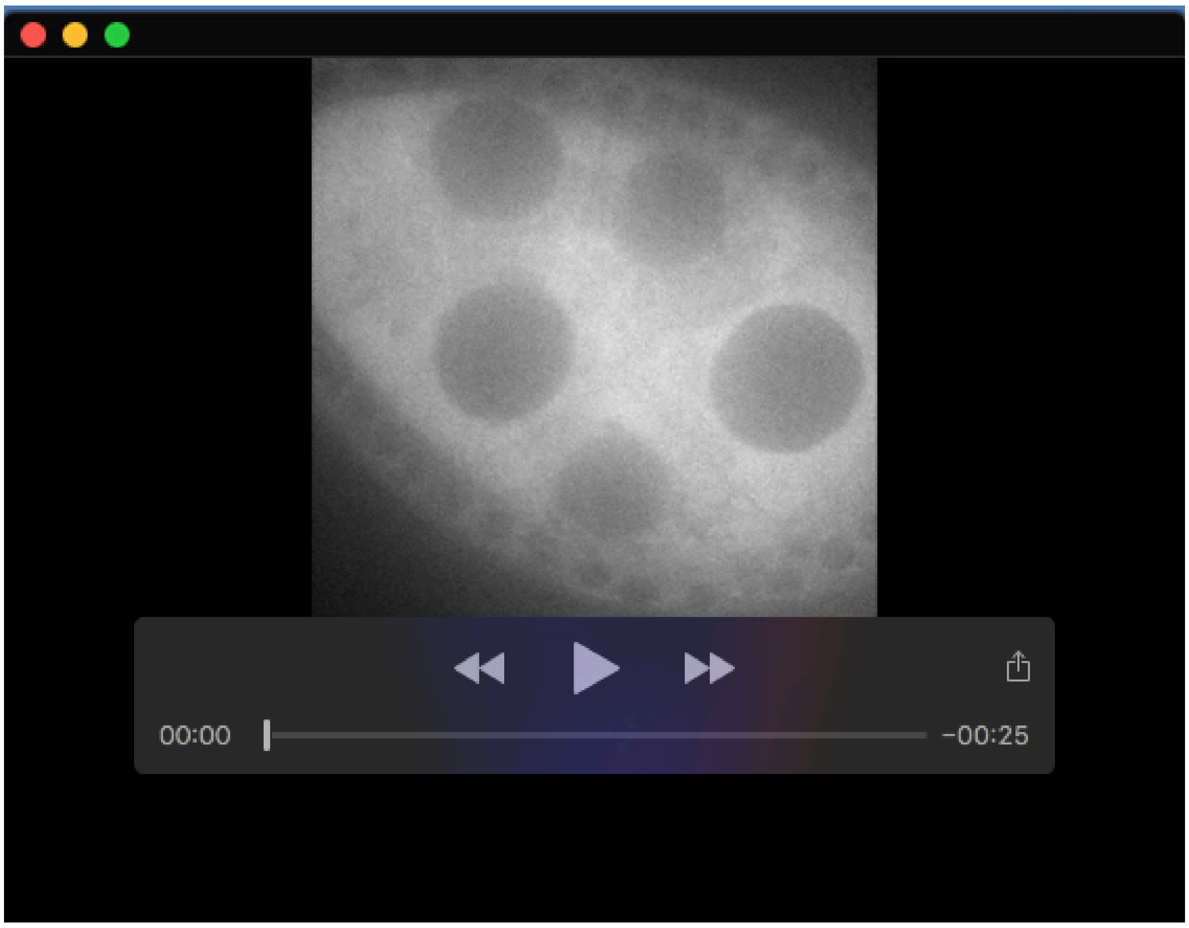

Movie 8. Live-imaging of the follicles from starved *clu*^{CA06604} female exposed to insulin. Dissected ovarioles from starved female were subjected to 100 µg/mL insulin. The object was recorded at 20-second intervals for 20 minutes using a Nikon Eclipse Ti2 spinning disk microscope. Video was recorded at ten frames per second.

Journal of Cell Science • Supplementary information

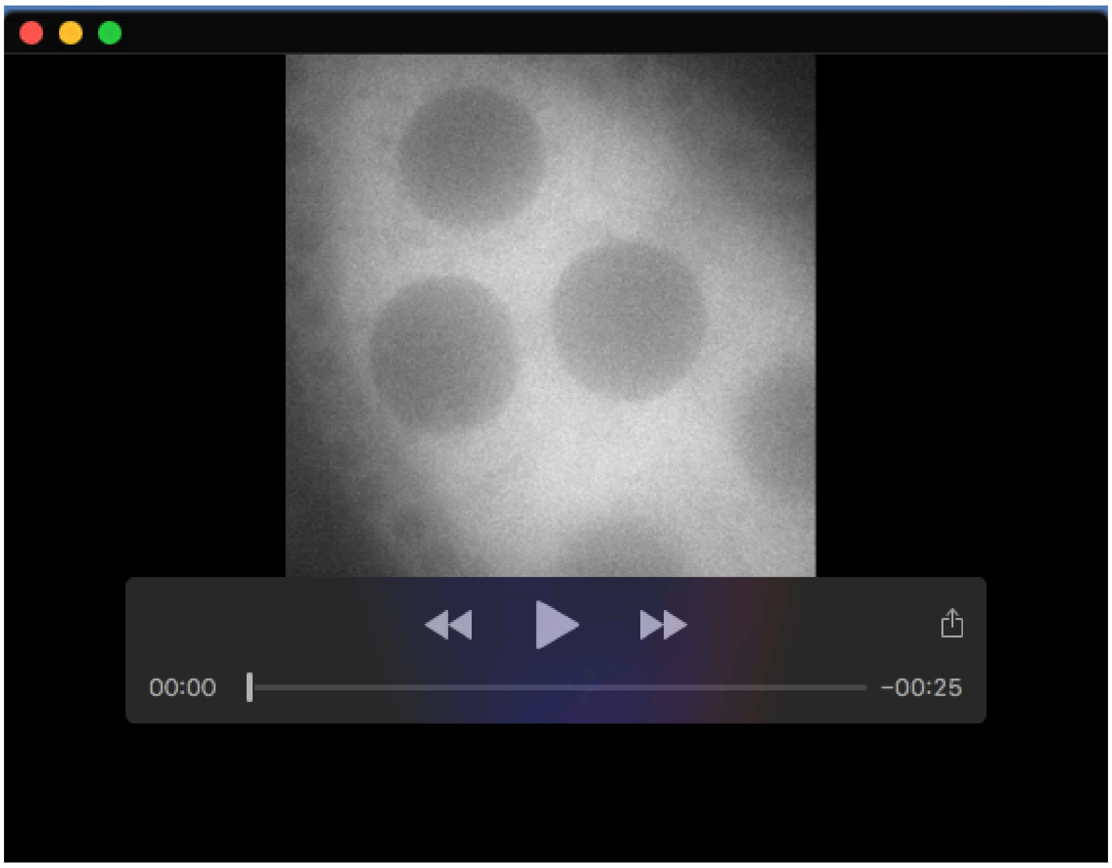

Movie 9. Live-imaging of the follicles from starved clu^{CA06604} female exposed to CHX followed by insulin. Dissected ovarioles from starved female were treated with CHX followed by 100 µg/mL insulin. The object was recorded at 20-second intervals for 20 minutes using a Nikon Eclipse Ti2 spinning disk microscope. Video was recorded at ten frames per second.

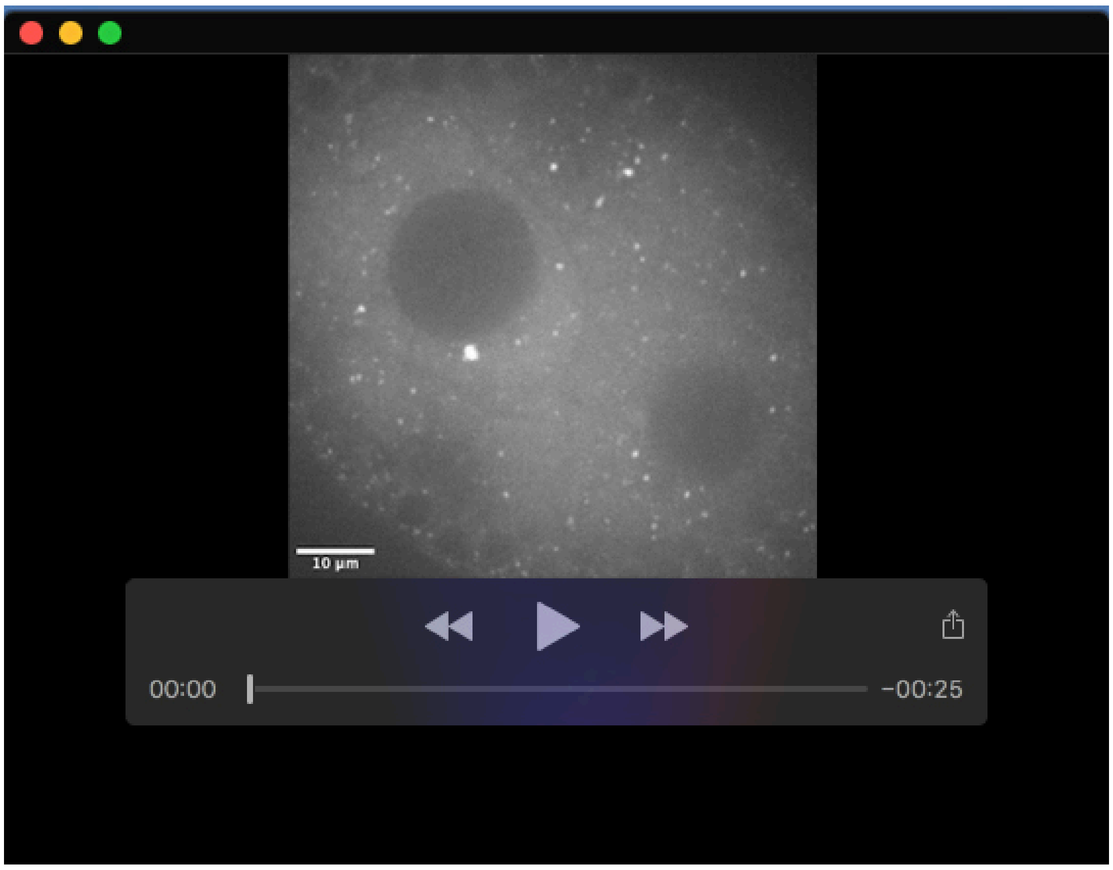

Movie 10. Live-imaging of the follicles from well-fed *clu*^{CA06604} female exposed to hydrogen peroxide after dissection with insulin-containing CS media. The object was recorded at 20-second intervals for 12 minutes using a Nikon Eclipse Ti2 spinning disk microscope. Video was recorded at ten frames per second.

Journal of Cell Science • Supplementary information

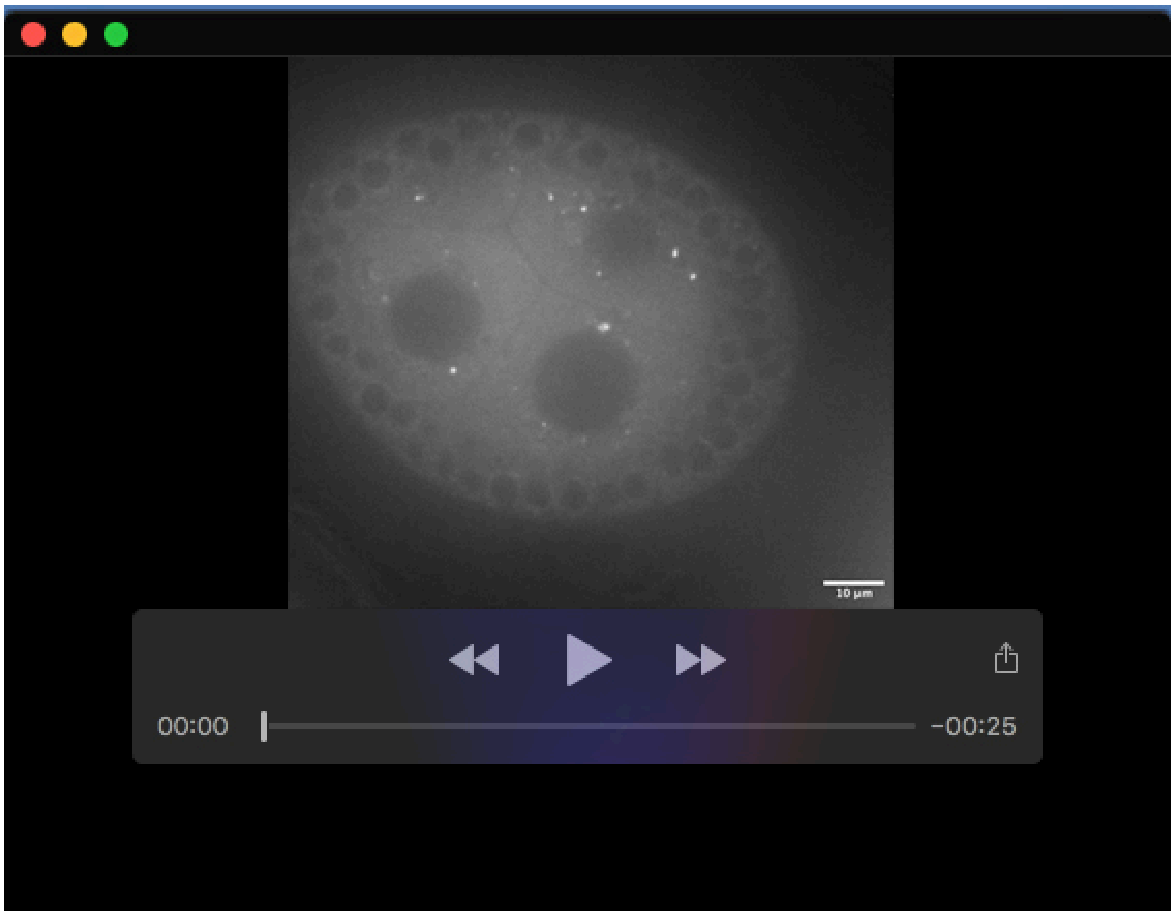

Movie 11. Live-imaging of the follicle from well-fed *clu*^{CA06604} female exposed to hydrogen peroxide following incubation with 3.5 mM CHX. The object was recorded at 20-second intervals for 12 minutes using a Nikon Eclipse Ti2 spinning disk microscope. Video was recorded at ten frames per second.

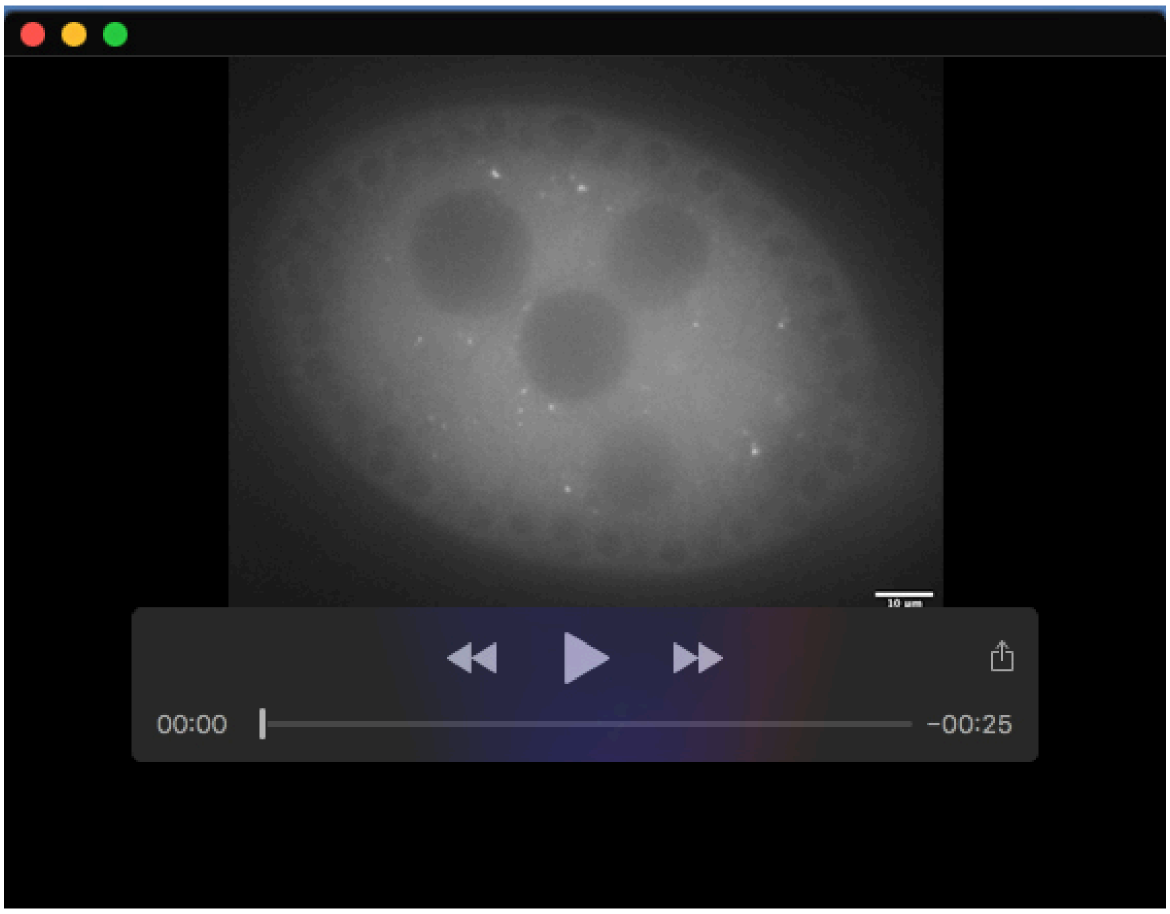

Movie 12. Live-imaging of the follicle from well-fed *clu*^{CA06604} female exposed to hydrogen peroxide following incubation with 7 mM CHX. The object was recorded at 20-second intervals for 12 minutes using a Nikon Eclipse Ti2 spinning disk microscope. Video was recorded at ten frames per second.
